# ActA Promotes *Listeria monocytogenes* Aggregation, Intestinal Colonization and Carriage

**DOI:** 10.1371/journal.ppat.1003131

**Published:** 2013-01-31

**Authors:** Laetitia Travier, Stéphanie Guadagnini, Edith Gouin, Alexandre Dufour, Viviane Chenal-Francisque, Pascale Cossart, Jean-Christophe Olivo-Marin, Jean-Marc Ghigo, Olivier Disson, Marc Lecuit

**Affiliations:** 1 Biology of Infection Unit, Institut Pasteur, Paris, France; 2 Inserm U1117, Paris, France; 3 Plateforme de Microscopie Ultrastructurale, Imagopole, Institut Pasteur, Paris, France; 4 Unité des Interactions Bactéries-Cellules, Institut Pasteur, Paris, France; 5 Inserm U604, INRA USC2020, Paris, France; 6 Unité Analyse d'Images Quantitative, Institut Pasteur, Paris, France; 7 CNRS URA 2582, Paris, France; 8 French National Reference Center and WHO Collaborating Center Listeria, Institut Pasteur, Paris, France; 9 Unité de Génétique des Biofilms, Institut Pasteur, Paris, France; 10 CNRS URA 2172, Paris, France; 11 Université Paris Descartes, Sorbonne Paris Cité, Centre d'Infectiologie Necker-Pasteur, Hôpital Universitaire Necker-Enfants Malades, Paris, France; Stanford University School of Medicine, United States of America

## Abstract

*Listeria monocytogenes* (*Lm*) is a ubiquitous bacterium able to survive and thrive within the environment and readily colonizes a wide range of substrates, often as a biofilm. It is also a facultative intracellular pathogen, which actively invades diverse hosts and induces listeriosis. So far, these two complementary facets of *Lm* biology have been studied independently. Here we demonstrate that the major *Lm* virulence determinant ActA, a PrfA-regulated gene product enabling actin polymerization and thereby promoting its intracellular motility and cell-to-cell spread, is critical for bacterial aggregation and biofilm formation. We show that ActA mediates *Lm* aggregation *via* direct ActA-ActA interactions and that the ActA C-terminal region, which is not involved in actin polymerization, is essential for aggregation *in vitro*. In mice permissive to orally-acquired listeriosis, ActA-mediated *Lm* aggregation is not observed in infected tissues but occurs in the gut lumen. Strikingly, ActA-dependent aggregating bacteria exhibit an increased ability to persist within the cecum and colon lumen of mice, and are shed in the feces three order of magnitude more efficiently and for twice as long than bacteria unable to aggregate. In conclusion, this study identifies a novel function for ActA and illustrates that in addition to contributing to its dissemination within the host, ActA plays a key role in *Lm* persistence within the host and in transmission from the host back to the environment.

## Author Summary

*Listeria monocytogenes* (*Lm*) is a ubiquitous bacterium that survives and thrives within the environment, and a facultative intracellular pathogen that induces listeriosis. So far, these two complementary facets of *Lm* biology have been studied independently. Here we identify ActA, which is a major *Lm* virulence determinant mediating actin-based motility, as critical for bacterial aggregation and biofilm formation. ActA promotes *Lm* aggregation *via* direct ActA-ActA interaction and ActA C-terminal region, which is not involved in actin polymerization, is essential for aggregation. Whereas ActA-mediated *Lm* aggregation is not observed in infected tissues, it occurs in the gut lumen. Strikingly, ActA-dependent aggregating bacteria exhibit an increased ability to persist within the gut lumen, and are shed in the feces three order of magnitude more and for twice as long than bacteria unable to aggregate. This study identifies a novel function for ActA, which plays a key role in *Lm* persistence within the host and transmission.

## Introduction

*Listeria monocytogenes* (*Lm*) is a facultative intracellular Gram-positive bacterium and the agent of listeriosis, the deadliest foodborne infection in humans, with a mortality rate between 20 to 30%. Listeriosis can manifest as gastroenteritis after ingestion of a high inoculum, as septicemia, meningitis and encephalitis primarily in immune-compromised individuals, and induce fetal-placental infection leading to *in utero* death, premature birth, abortion and neonatal infection.

*Lm* induces its internalization in non-professional phagocytes, such as epithelial cells, survives and multiplies in the cytosol of professional phagocytes and spreads from cell to cell. These properties constitute crucial virulence determinants of *Lm* and their molecular mechanisms have been studied in detail. InlA and InlB have been identified as critical surface proteins mediating *Lm* entry into epithelial cells [1], [2] and crossing of the intestinal and placental barriers [3]–[6]. Listeriolysin O (LLO) is a pore-forming toxin that mediates *Lm* escape from the internalization vacuole, and its access to the cytosol [7]. It is a critical phenotypic marker for *Lm* identification and is the virulence factor that allows *Lm* survival in professional phagocytes [8]. Once in the cytosol, *Lm* polymerizes actin to propel itself, forming protrusions at the host cell surface and spread from cell to cell. ActA has been identified as the *Lm* factor necessary and sufficient on the bacterial side to polymerize actin and form comet tails [9]. Recently, ActA has also been shown to allow *Lm* to escape autophagy [10]. PrfA, a transcriptional activator that belongs to the cyclic AMP receptor protein family regulates most genes involved in *Lm* virulence, including *inlA*, *inlB*, *hly* (which encodes LLO) and *actA* [11]–[13]. PrfA is expressed during *Lm* exponential growth and at the beginning of stationary phase [12], above 30°C [11]. This key regulator is selectively activated *in vivo* in the intestinal lumen, enabling *Lm* to switch on its virulence genes [14]. PrfA is specific to the pathogenic species *Lm* and *L. innocua* (*Li*), a non-pathogenic non-invasive *Listeria* species closely related to *Lm*, is devoid of PrfA and PrfA-regulated genes, including *inlA*, *inlB*, *hly* and *actA* [1].

Because *Lm* is primarily regarded as a pathogen, its pathogenicity is the aspect of its biology that has been studied in the most detail. Nevertheless, *Lm* can be shed asymptomatically, persist in human and animal feces and be released in the environment [15], [16]. *Lm* is a ubiquitous bacterium that also thrives in diverse external environments such as soil, water, decaying plants, and silage, exposing wild animals and cattle to multiple opportunities of ingestion and perpetuating *Lm* transmission [17]. In the environment, bacteria can form biofilms, which favor their persistence [18]. From a food-safety perspective and with the aim of limiting *Lm* transmission to humans, a lot of emphasis has been focused on reducing bacterial aggregation, biofilm formation and persistence of *Lm* on industrial surfaces and food [19]. A number of factors, including the quorum-sensing-related proteins of the LuxS and Agr systems [20], [21], and stress responses factors [22]–[25] such as the transcriptional regulator SigB [26], and PrfA [27], [28] have been implicated in *Lm* biofilm formation. Yet, neither *Lm* persistence nor the putative role of bacterial aggregation and biofilm formation has been investigated in the context of infection,

Our study began with the serendipitous observation that *Lm* spontaneously sediments in test tubes whereas *Li* does not. Fast sedimentation is usually triggered by tight interactions mediated by aggregation factors generally involved in biofilm formation [29], [30]. We show here that *Lm* rapid sedimentation results from PrfA-dependent aggregation. Furthermore, we show that ActA is the PrfA-regulated factor promoting bacterial aggregation *via* direct ActA-ActA interaction. Finally, we show that ActA-dependent bacterial aggregation leads to increased *Lm* persistence in the intestine, prolonged fecal shedding and thereby facilitates transmission. This is a critical new function for ActA, which manifests extracellularly, and is independent of its role in actin-based motility. Virulence factors may confer a selective advantage for pathogenic microbes, when they allow the colonization of otherwise sterile host tissues. This newly observed property of ActA may also participate in the selective pressure on *Lm* to maintain ActA, as it favors bacterial dissemination.

## Results

### *Listeria monocytogenes* forms aggregates in a PrfA-dependent manner

**Figure 1:**
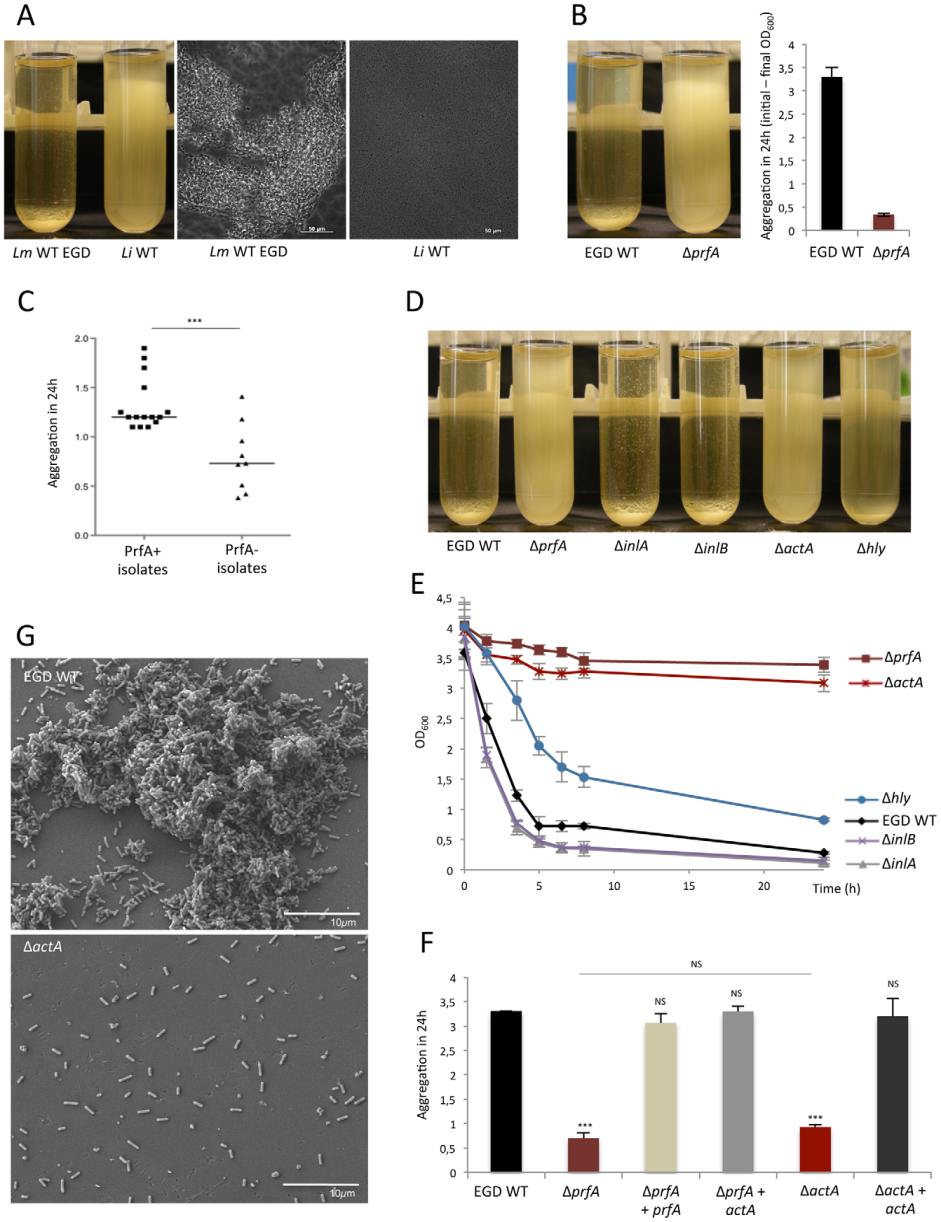
*actA*, a PrfA-regulated gene, mediates *Lm* aggregation. (**A**) Aggregation assay performed on EGD WT (*Lm*) strain and *L. innocua (Li)* (left panel) in BHI and observation by bright field microscopy of the bacteria that have sedimented (right panel). Scale: 50 µm. (**B**) Aggregation assay realized using EGD WT and Δ*prfA* in BHI and quantification of their aggregation ability in 24 h by subtracting the measured final OD_600_ to the initial OD_600_. (**C**) Comparison of aggregation abilities of *Lm* PrfA+ isolates *versus prfA*-mutated strains PrfA−, from National Reference Center. Aggregation assay was realized in DMEM to increase PrfA-regulated genes expression. (**D**) Aggregation assay for five hours and (**E**) over time performed on EGD WT and its isogenic deletion mutants for *prfA*, *inlA*, *inlB*, *hly* and *actA* genes, in BHI. (**F**) Aggregation assay in BHI of EGD WT, Δ*prfA*, the complemented mutants Δ*prfA* + *prfA* and Δ*prfA* + *actA*, Δ*actA* and the complemented strain Δ*actA + actA*. (**G**) Observation of EGD WT and Δ*actA* by SEM after aggregation assay. Scale: 10 µm.

When *Lm* (EGD strain) and *Li* cultures grown overnight in BHI, at 37°C with shaking, were switched to static conditions, *Lm* EGD sedimented within five hours whereas *Li* did not (Figure 1A). Microscopic examination of the pellet revealed bacterial aggregates (Figure 1A) and this phenotype was abolished when *Lm* was grown at 25°C (data not shown). Because *Li* lacks PrfA and PrfA-regulated genes, which are specific to *Lm* and regulated by temperature, we investigated whether *prfA* could be implicated in *Lm* aggregation. An aggregation assay performed with EGD and an isogenic mutant Δ*prfA* showed that aggregation is *prfA*-dependent (Figure 1B). Similar results were observed for the other *Lm* reference strains, LO28 and EGDe, when cultivated in BHI (Figures S1A–B) or in DMEM (Figures S1C–D), in which PrfA-regulated genes expression and the aggregation phenotype were increased [31].

To confirm the role of *prfA* in *Lm* aggregation, we performed aggregation assay with clinical strains, randomly chosen from the collection of the French National Reference Center for *Listeria* and harboring a functional PrfA (PrfA+), and non-clinical isolates, naturally non-hemolytic and lacking phospholipase activity due to loss-of-function of PrfA (PrfA−) (Table S1) (our unpublished observations). PrfA expression by both PrfA+ and PrfA− isolates was confirmed by immunoblot (data not shown). The mean aggregation in 24 h of the PrfA− isolates was significantly reduced (p = 0.001) when compared to the mean aggregation of PrfA+ (Figure 1C), showing that the role of *prfA* in *Lm* aggregation is a general property of various *Lm* strains.

### ActA is the PrfA-regulated factor involved in *Listeria monocytogenes* aggregation

To determine how PrfA regulates *Lm* aggregation, we analyzed isogenic deletion mutants of the main PrfA-regulated virulence genes, *i.e. inlA*, *inlB*, *hly* and *actA*. Δ*inlA* and Δ*inlB* EGD isogenic mutants displayed an ability to aggregate identical to that of WT EGD and the aggregation ability of Δ*hly* mutant was marginally delayed compared to the WT (Figures 1D–E). In contrast, both Δ*prfA* and Δ*actA* mutants displayed very low aggregation, even after 24 h (Figures 1D–E). Consistent with these results, complementation of Δ*prfA* and Δ*actA* mutants either with *prfA* or *actA* fully restored WT aggregation ability (Figure 1F). Similar results were obtained with LO28 and EGDe strains (Figures S1E–F). Observation by scanning electron microscopy (SEM) of WT *Lm* sediment retrieved after five hours under static conditions showed dense bacterial aggregates, whereas no aggregate was detected with the Δ*actA* mutant (Figure 1G). Together, these results demonstrate that ActA is the PrfA-regulated gene product involved in the formation of *Lm* aggregates.

### ActA is involved in *Listeria monocytogenes* biofilm formation

**Figure 2:**
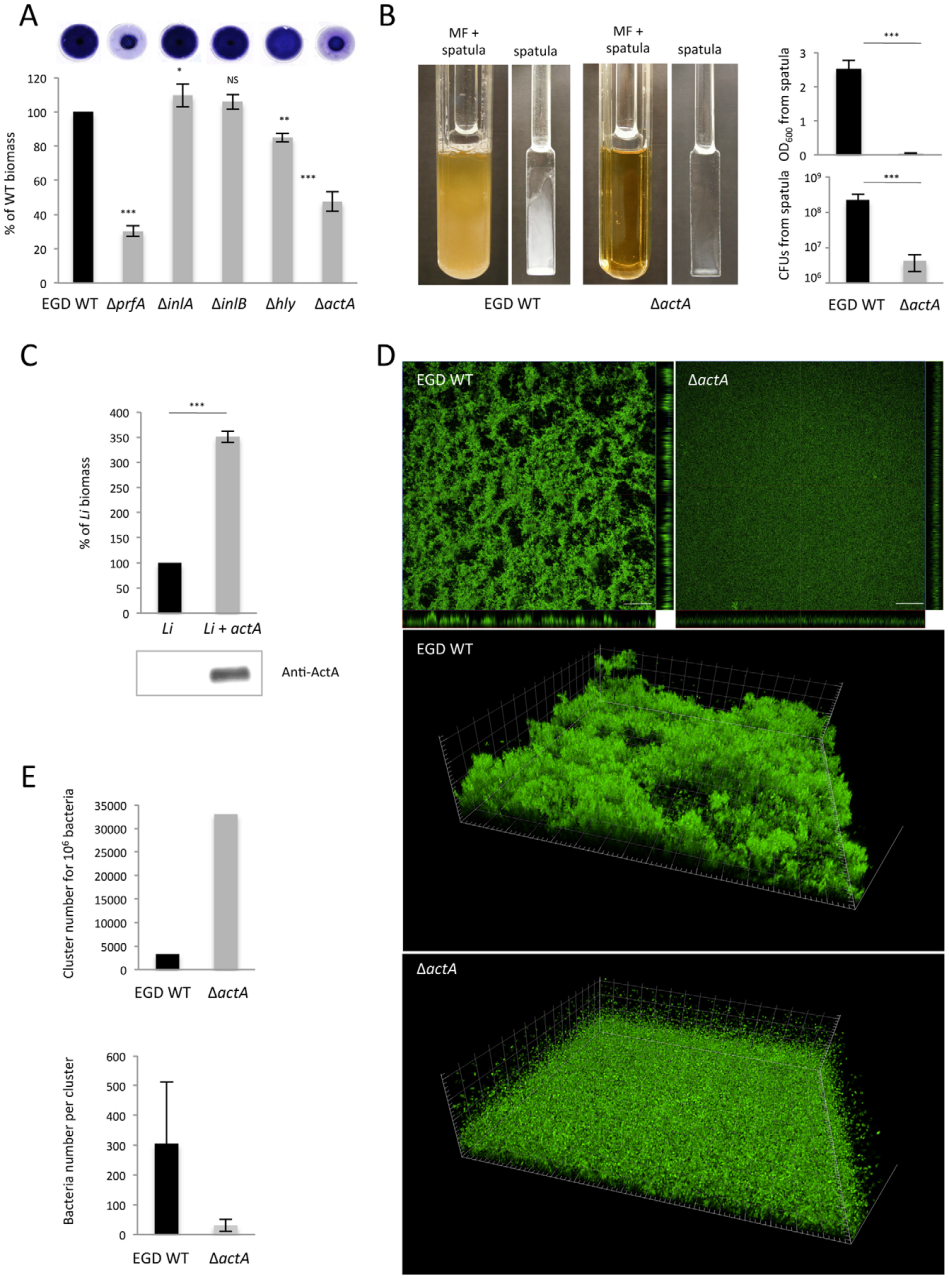
ActA mediates *Lm* biofilm formation. (**A**) Microtiter-plate biofilm assay of EGD WT and the isogenic deletion mutants for *prfA*, *inlA*, *inlB*, *hly* and *actA* genes. Biofilm biomass formed in 24 h at the surface of the wells and stained by crystal violet (bottom view of the wells) was quantified by densitometry. (**B**) Comparison of WT *versus* Δ*actA* mature biofilm formed in continuous-flow microfermentors in 42 h. Biofilm biomasses formed on spatula were quantified by spectrophotometry and by CFUs enumeration. (**C**) Comparison of biofilms formed in microtiter-plate in 24 h after expression of *actA* in *Li*. (**D**) Observation of EGD WT *versus* Δ*actA* biofilms formed on glass slides by confocal microscopy. Scale: top views, 100 µm; side views, 20 µm per square. (**E**) Quantification of clusters formed by EGD WT *versus* Δ*actA* in biofilms. Cluster number for 10^6^ bacteria as well as the mean number of bacteria per cluster were calculated.

As bacterial aggregation is a key step of biofilm formation [18], we investigated the contribution of ActA to *Lm* biofilm formation *in vitro* with EGD isogenic mutants Δ*prfA*, Δ*inlA*, Δ*inlB*, Δ*actA* and Δ*hly*. Whereas biofilm biomass of WT EGD could be homogenously and strongly stained by crystal violet on the surface of the wells, the Δ*prfA* mutant displayed a 70% reduction in biofilm biomass, which was only present in the center of the wells (Figure 2A). Δ*inlA* formed slightly but significantly more biofilm than WT, Δ*inlB* was equivalent to WT and Δ*hly* formed slightly less biofilm as compared to WT (Figure 2A). In contrast, Δa*ctA* displayed 55% biofilm reduction and was the only strain impaired in covering the bottom of wells as observed for Δ*prfA* (Figure 2A). This suggests that ActA is the major PrfA-regulated gene involved in biofilm formation.

To confirm the involvement of ActA in biofilm formation, we used continuous-flow microfermentors. Whereas WT biofilm grew on both spatula and microfermentor walls, Δ*actA* exhibited a drastically reduced ability to form biofilm (Figure 2B). Comparisons of the biomass retrieved from biofilms formed on the spatula between the WT and the isogenic Δ*actA* mutant showed a 60-fold difference in optical density at 600 nm (OD_600_) and a reduction of two orders of magnitude in CFUs (Figure 2B).

To determine if other factors are required to trigger ActA-dependent biofilm formation, we expressed *actA* in *Li*, which only forms a very limited biofilm biomass in microtiter plate. ActA expression in *Li* + *actA* was confirmed by immunoblot and immunofluorescence (Figure 2C and data not shown). Biofilm assay in microtiter-plate showed a significant increase of biomass following the expression of *actA* by *Li* (Figure 2C), indicating that ActA is sufficient to promote biofilm formation in *Li*.

We next imaged EGD WT and Δ*actA* grown on static glass slide by confocal microscopy. Whereas the Δ*actA* bacteria organized in a very thin and homogenous layer around 25 µm thick, the WT formed a deep mushroom-shaped and dense biofilm around 45 µm thick (Figure 2D). For an equivalent number of bacteria, there were one order of magnitude fewer WT clusters than with Δ*actA*, and the number of bacteria per cluster with WT bacteria was one order of magnitude higher than with Δ*actA* (Figure 2E). Taken together, these data show that bacteria expressing ActA aggregate into large clusters within biofilm structure thereby favoring biofilm formation, which is not the case for Δ*actA*.

### A direct ActA-ActA interaction mediates *Lm* aggregation

**Figure 3:**
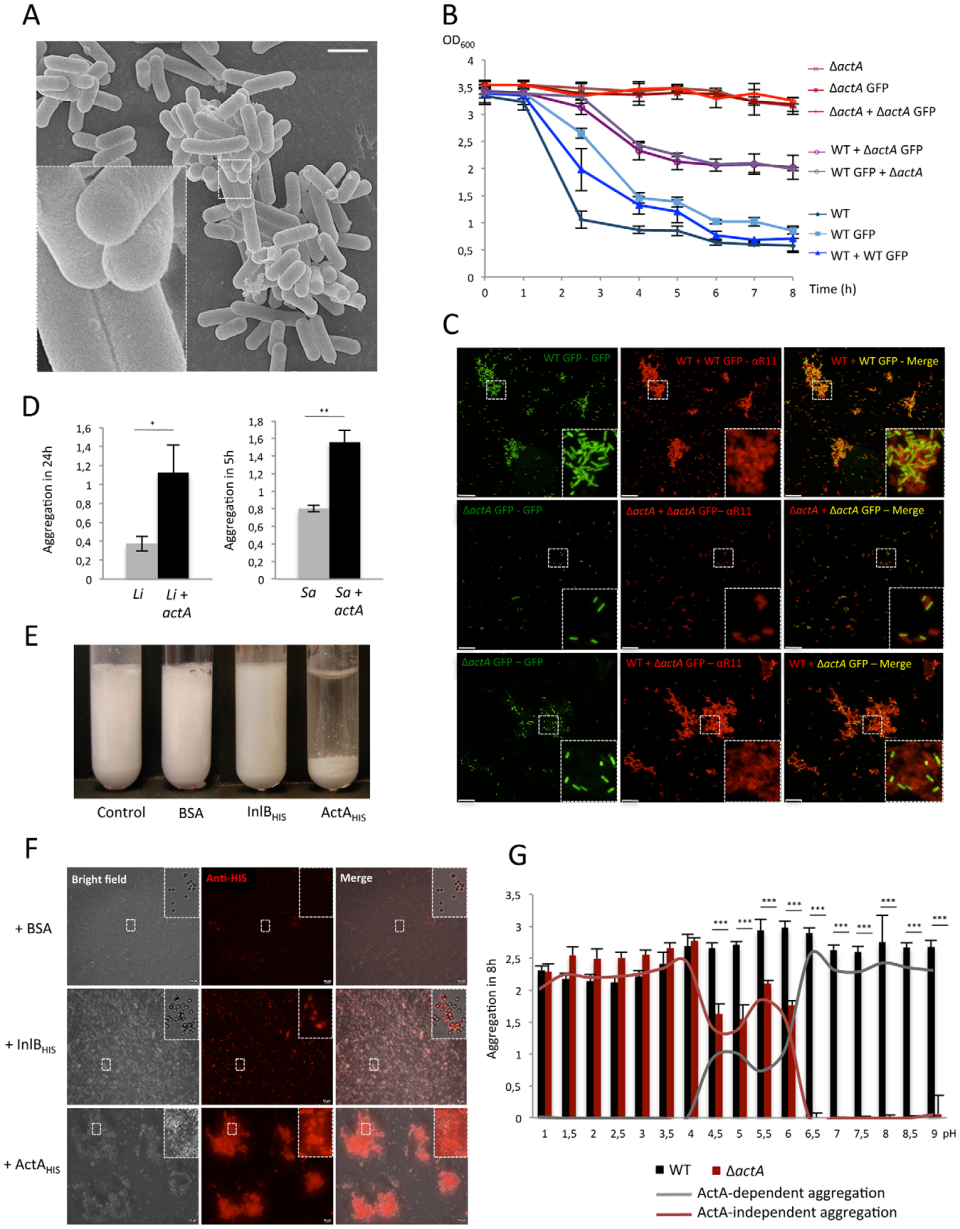
Direct ActA-ActA interaction mediates aggregation. (**A**) Observation of EGD aggregates by SEM. Scale: 1 µm. (**B**) Aggregation assay performed mixing EGD WT +/− GFP and Δ*actA* +/− GFP mutants. (**C**) Observation by immunofluorescence on confocal microscope of bacteria collected from bottom of the tubes after mixed aggregation assay of WT +/− GFP and Δ*actA* +/− GFP bacteria. Mixed bacteria populations were detected using GFP (green) and staining with anti-*Lm* R11 antibody (red). Scale: 10 µm. (**D**) Aggregation assay performed with *Li* and *S. aureus* expressing *actA*. (**E**) Latex beads aggregation assay realized by mixing beads with BSA or purified InlB_HIS_ or ActA_HIS_ proteins. (**F**) Observation by immunofluorescence of beads collected during latex beads aggregation with BSA, InlB_HIS_ or ActA_HIS_. Coupling of InlB_HIS_ and ActA_HIS_ on beads was assessed using anti-HIS antibody (red). Scale: 10 µm. (**G**) Aggregation assay comparing aggregation ability of EGD WT and Δ*actA* in different BHI media of pH ranging from 1 to 9.

ActA is a membrane-anchored protein exposed on the bacterial surface [9]. Either direct or indirect ActA-ActA interaction may mediate bacterial aggregation and favor biofilm formation. We observed that ActA-dependent aggregation occurs in PBS and H_2_O (data not shown), suggesting that external factors are not required for *Lm* aggregation. Moreover, when observed by SEM, bacterial aggregates did not exhibit visible matrix connecting bacteria to each other, suggesting that ActA-dependent aggregation occurs without any incorporation of matrix (Figure 3A).

In order to determine whether aggregation is mediated by a direct ActA-ActA interaction, we performed aggregation assays by mixing EGD WT and/or Δ*actA* bacteria expressing green fluorescent protein (GFP) or not. As expected, WT and WT GFP formed mixed aggregates (Figure 3B–C). In contrast, Δ*actA* and Δ*actA* GFP did not aggregate (Figure 3B), and only constituted small and isolated mixed bacterial foci (Figure 3C, two top rows). In the case of mixed WT and Δ*actA* GFP bacteria, we observed an intermediate aggregation phenotype and aggregates contained almost exclusively WT bacteria, with some sparse Δ*actA* GFP bacteria trapped within the aggregative structure (Figure 3B–C). These results show that Δ*actA* bacteria are not able to aggregate with WT, and suggest that ActA-dependent aggregation requires a direct ActA-ActA interaction.

To study whether ActA is sufficient to promote *Lm* inter-bacterial interactions, aggregation assays were performed with ActA-expressing *Li* and *Staphylococcus aureus* strains. We observed that ActA expression is sufficient to promote the aggregation of these two strains (Figure 3D). Finally, we performed an aggregation assay with latex beads coated with purified ActA_HIS_, InlB_HIS_ or bovine serum albumin (BSA) [32], [33], [34]. The coating of beads was assessed by immunofluorescence and a strong signal corresponding to either ActA_HIS_ or InlB_HIS_ coated on beads was detected (Figure 3F). The aggregation assays showed that ActA_HIS_-cotaed latex beads formed macroscopic aggregates within 15 minutes (Figure 3E–F). In contrast, latex beads coated with either BSA or purified InlB_HIS_ did not, even after 24 hours. Together, these data demonstrate that direct ActA-ActA interaction mediates aggregation.

ActA has a low isoelectric point (pI of 4.95), indicating that ActA-dependent aggregation at neutral pH, at which our experiments were performed, occurs when ActA is globally negatively charged. We hypothesized that ActA charge could be important for aggregation and performed aggregation assays in a pH range of 1 to 9. Whereas overall bacterial aggregation within this pH range was roughly stable, ActA-mediated aggregation was maximal between pH 6.5 to pH 9, a pH window within which no ActA-independent aggregation is detected (Figure 3G).

### *Listeria* aggregation requires the expression of full-length ActA

**Figure 4:**
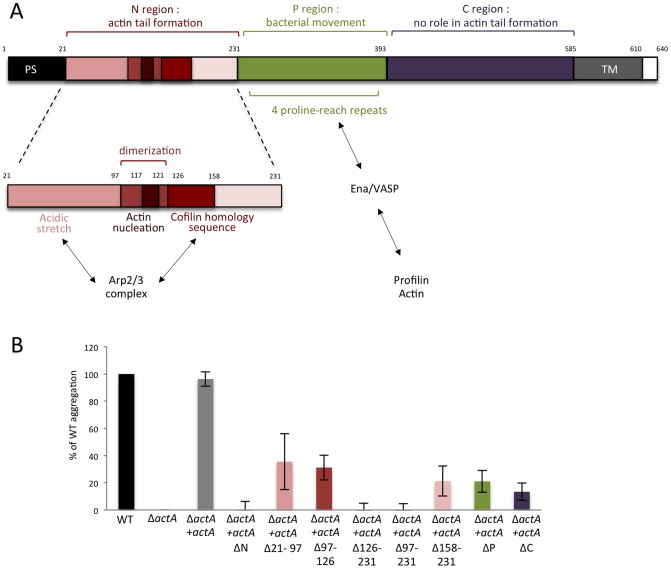
Role of ActA domains in aggregation. (**A**) Roles of the ActA domains in cell-to-cell spread. ActA is composed of 640 amino acids (AA). It harbors a 21AA-signal peptide and a transmembrane domain (AA585–610) close to the COOH-terminal domain. According to the nomenclature proposed by Lasa *et al.* in 1997 [36], the NH2-terminal domain or N region (AA21–231) is essential for host actin polymerization [35], [37], especially the regions 117–121 and 126–158 [36]. However, the N region does not directly stimulate actin polymerization but rather mediates actin nucleation with the Arp2/3 complex [32]. Arp2/3 complex is recruited *via* a basic cofilin homology sequence within the 126–158 region and an acidic stretch within 21–97 domain [39]. This latter helps for maintenance and continuity of filament elongation. Region 97–126 delimits also a putative dimerization domain [50]. The central P region (AA232–393) contains four proline-rich repeats that bind to Enabled/vasodilator-stimulated phosphoprotein (Ena/VASP) family proteins [36], which in turn bind to actin filaments and the actin-binding protein profilin [40]. The P region is involved in bacteria movement modulating length of comet tails [35], [37]. C region (AA394–585) is not implicated in cell-to-cell spread process [36]. (**B**) Aggregation assay performed on LO28 Δ*actA* mutant complemented with different truncated forms of the different *actA* domains.

To further investigate how ActA mediates *Lm* aggregation, we functionally mapped the ActA domains involved in bacterial aggregation. The respective contribution of ActA domains in host actin polymerization have been previously determined. These studies have shown that (*i*) the NH2-terminal domain (N region) binds Arp2/3 complex, is involved in actin filament nucleation and is critical for actin polymerization, (*ii*) the central domain (P region) binds Ena/VASP, is not required for actin polymerization but contributes to the length of actin tails and the velocity of bacterial intracellular movement, and (*iii*) the C-terminal or C region is dispensable for actin polymerization [32], [35]–[43] (Figure 4A).

When aggregation assays were performed with strains expressing ActA variants lacking the N, P or C region, or subdomains within the N region (Figure S2B), we observed that only full-length ActA mediates full aggregation, suggesting that aggregation requires the native conformation of the full-length ActA protein. We also observed that the consecutive 21–97 and 97–126 segments in N-region were only partially implicated in aggregation, allowing 31% and 36% of aggregation, respectively. In contrast, the 126–231 segment of N-region appeared critical for aggregation (Figure 4B). Both mutants lacking P and C regions were also impaired in aggregation.

### ActA promotes *Listeria* aggregation within gut lumen

**Figure 5:**
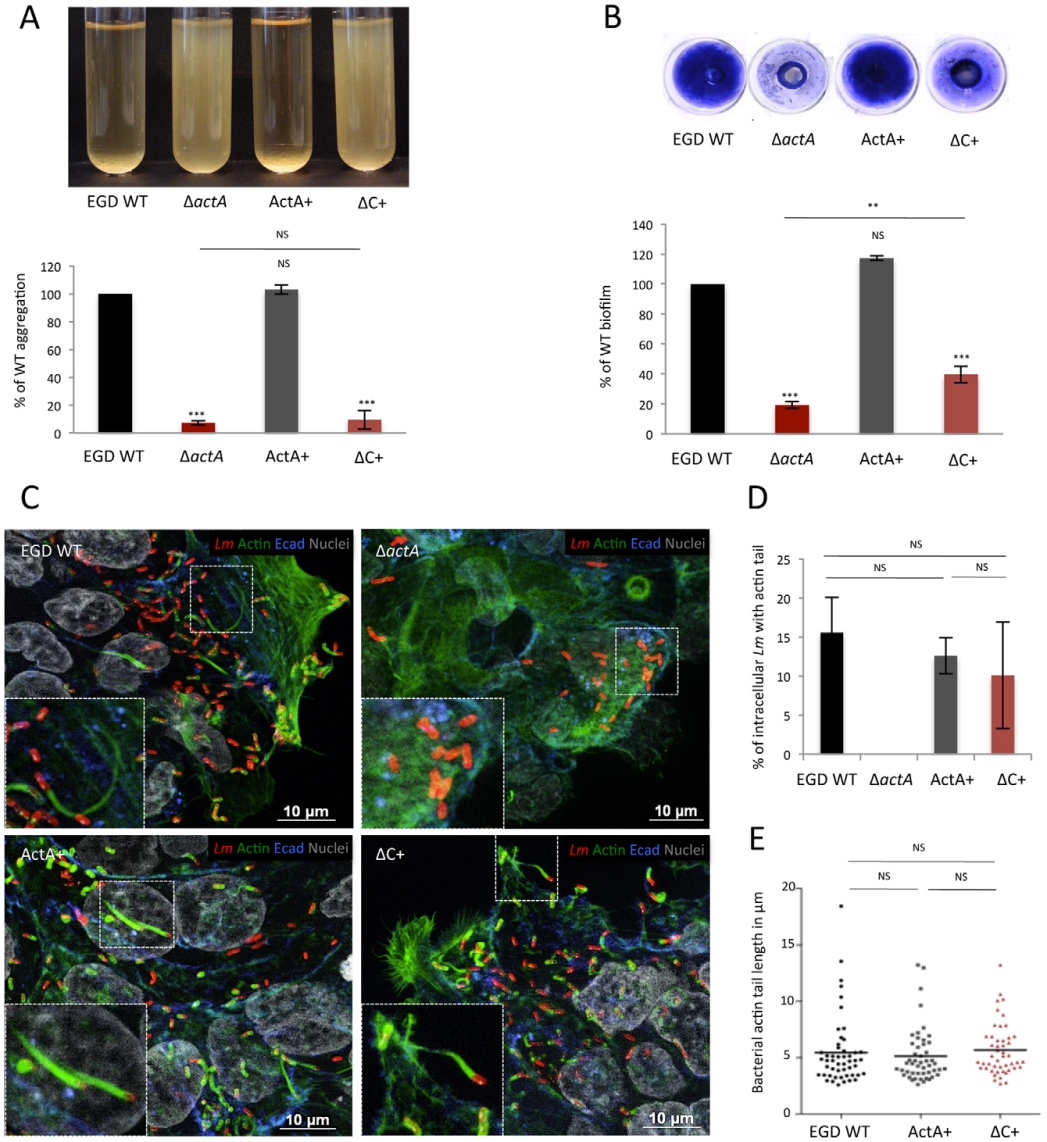
ActA C-region, which is dispensable for actin-based motility, is involved in aggregation. Comparison of aggregation and biofilm formation abilities of EGD WT, Δ*actA* and both Δ*actA* complemented-mutants with *actA* (ActA+) or *actA*ΔC (ΔC+) by aggregation assay (**A**) and microtiter-plate biofilm assay (**B**), respectively. (**C**) Ability of these mutants to polymerize host actin was compared after invasion assay on T84 cells. Bacteria were detected with anti-*Lm* (red), bacteria actin tails and host actin using phalloidin (green) whereas cells were delimited with an anti-Ecadherin (Ecad, blue) antibody and nuclei labeled with Hoechst (grey). Scale: 10 µm. (**D**) Comparison of the percentage of intracellular bacteria harboring an actin tail after T84 invasion assay. (**E**) Mean length of actin tails formed by intracellular bacteria after T84 invasion assay.

Because the C-terminal region of ActA, which is not involved in actin polymerization, is implicated in aggregation, we took advantage of this property to directly assess the contribution of ActA-dependent aggregation during infection, independent of the critical role of ActA in actin-based motility. To this aim, we complemented EGD Δ*actA* mutant with a C-region-truncated *actA*. We first confirmed that the EGD Δ*actA* + *actA*ΔC (ΔC+) strain was impaired in its abilities to either aggregate or form biofilm, as is the Δ*actA* mutant (Figures 5A–B). We also checked the ability of the ΔC+ mutant to polymerize actin in cultured cells. We observed that ΔC+ bacteria formed actin comet tails as efficiently as WT and Δ*actA* + *actA* (ActA+) (Figure 5C). Furthermore, ΔC+ intracellular bacteria were able to induce comet tails as WT and ActA+ (Figure 5D) and ΔC+ comet tails were of similar length than that of WT and ActA+ (Figure 5E). These results showed that ΔC+ mutant phenotype is similar to that of WT and ActA+, as far as actin-based motility is concerned, but is impaired for biofilm formation and aggregation like Δ*actA*.

**Figure 6:**
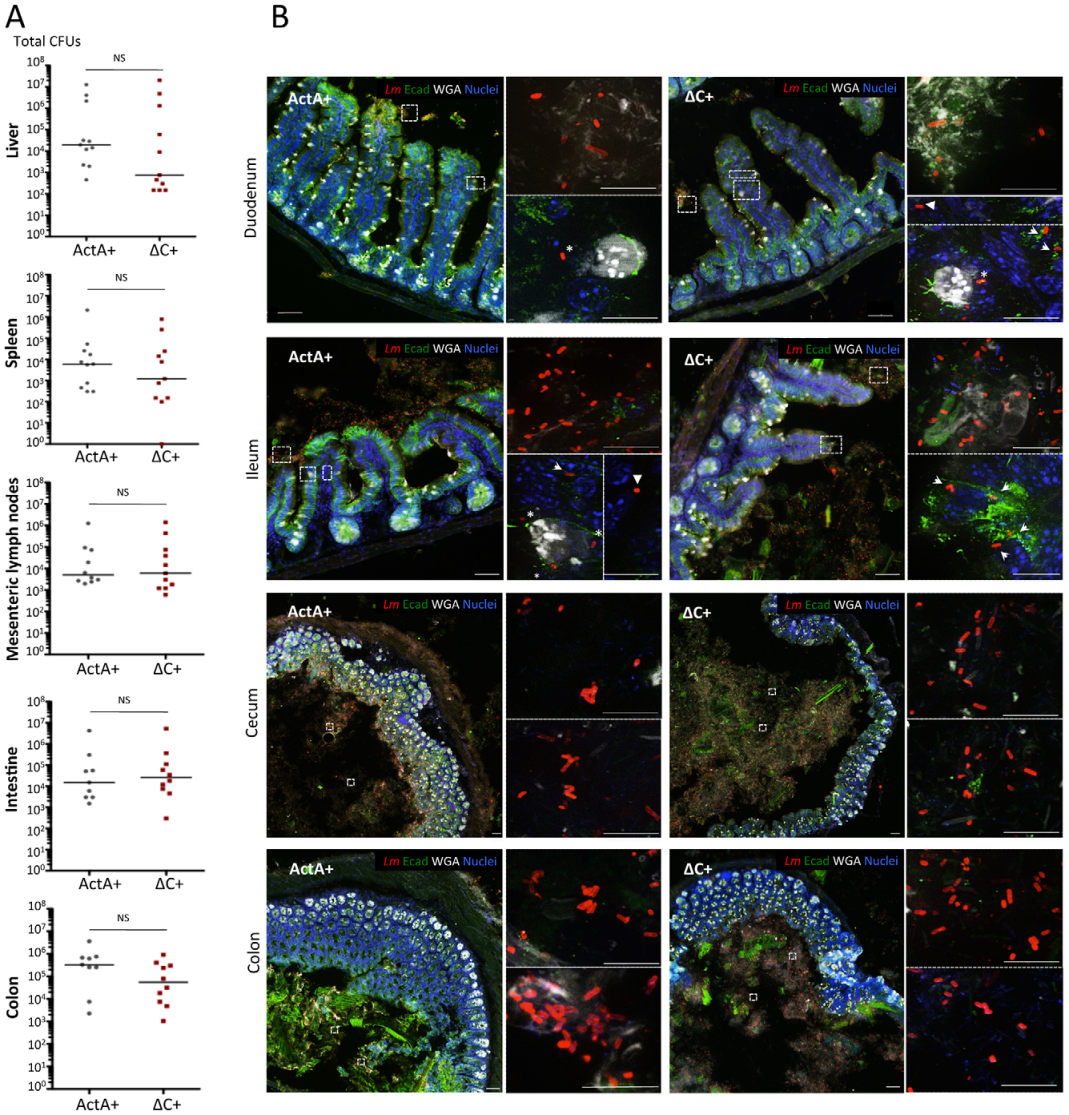
ActA C-region is involved in aggregation but not in tissue invasion. (**A**) Virulence of EGD Δ*actA* + *actA* (ActA+) and EGD Δ*actA* + *actA*ΔC (ΔC+) strains is evaluated by enumerating CFUs contained within mice liver, spleen, mesenteric lymph nodes, intestine and colon, four days after oral infection of KI E16P mice. (**B**) Observation of whole duodenum, ileum, cecum and colon of mice infected with either ActA+ or ΔC+ strains, six hours post-infection. Bacteria were labeled with anti-*Lm* (red) whereas intestinal epithelial cells were detected using an anti-E-cadherin (Ecad, green), mucus and cell membrane with WGA (white) and Hoechst (blue). Star: bacteria within goblet cells. Arrow: bacteria within extruding cells or epithelial folds. Arrowhead: bacteria within *lamina propria*. Scale of low magnification pictures: 50 µm. Scale of insets: 10 µm.

We next inoculated knock-in humanized E16P mEcad (KI E16P) mice, which are permissive to orally-acquired listeriosis, with either EGD ActA+ or ΔC+ strains, to investigate the role of ActA-dependent aggregation *in vivo*, independent of the critical role of ActA in actin-based motility [4]. Four days after inoculation, no significant difference in CFU counts in the intestine and colon tissues, mesenteric lymph nodes, spleen and liver were detected (Figure 6A). This result shows that both ActA+ and ΔC+ are similarly invasive *in vivo*, and consequently that the ability of ActA to mediate *Lm* aggregation does not have an impact on *Lm* ability to infect tissues in the first four days of infection.

We next investigated if ActA-mediated aggregation occurs within the intestine, which pH is >6.5, except in the stomach and proximal duodenum, and therefore optimal for ActA-mediated aggregation. We first checked that ActA is expressed within the gut lumen (Figure S2C). We then performed a detailed imaging survey for bacteria within the whole small intestine, cecum and colon six hours after oral inoculation. For both EGD ActA+ and ΔC+ strains, we observed rare isolated bacteria within the duodenal and ileal lumens, which fits with the rapid transit of *Lm* in the small intestine upon oral inoculation ([44], [45] and our unpublished observations). Isolated intracellular bacteria were also found within the intestinal epithelium, and particularly in goblet cells, extruding cells and epithelial folds, which are the preferential sites for *Lm* entry within the intestine [3], [6], [46]. Bacteria were also observed within the *lamina propria* of intestinal villi, confirming that both mutants are equally invasive (Figure 6A–B). Importantly, within the cecum lumen, ActA+ and ΔC+ strains exhibited distinct phenotypes as early as six hours post-inoculation: whereas ΔC+ bacteria remained mainly isolated, ActA+ bacteria formed small aggregates. This distinctive phenotype was also observed within the colon lumen, in which ActA+ bacteria aggregates were detected, often trapped within mucus, whereas none was observed with ΔC+ (Figure 6B). Together, these results show that ActA-dependent aggregation is detectable *in vivo* in the cecum lumen as early as six hours post inoculation.

**Figure 7:**
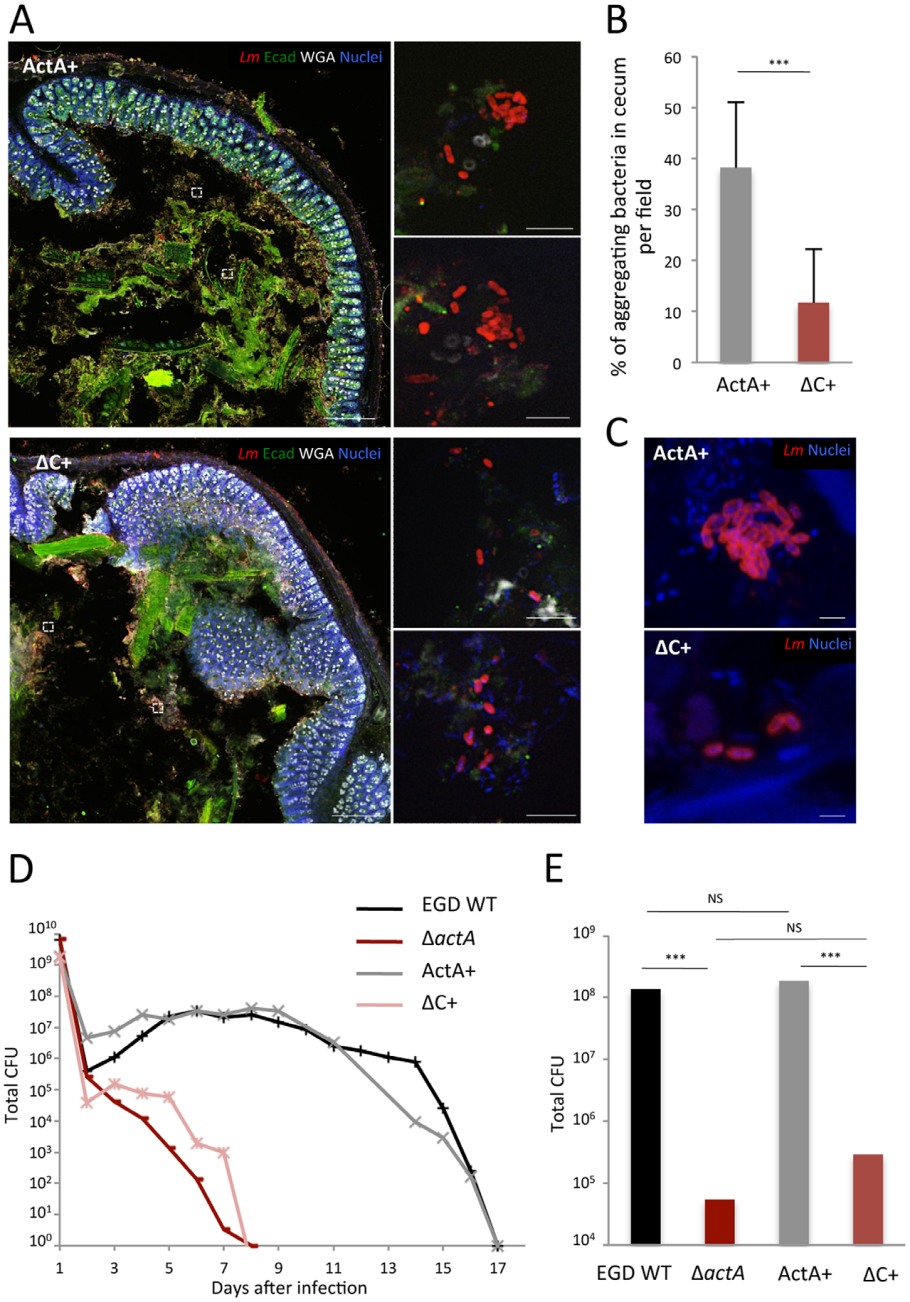
ActA-dependent aggregation and *Lm* colonization of the gut. (**A**) Observation of cecum of mice infected with either EGD Δ*actA* + *actA* (ActA+) and EGD Δ*actA* + *actA*ΔC (ΔC+) strains, 96 h post-infection. Bacteria were labeled with anti-*Lm* (red); intestinal epithelial cells were detected using anti-E-cadherin (Ecad, green), mucus and cell membrane with WGA (white) and DNA (nuclei) with Hoechst (blue). Scale of low magnification pictures: 100 µm. Scale of insets: 5 µm. (**B**) Percentage of aggregating bacteria within cecum lumen per optical field observed in (A). At least three bacteria in interaction defined the minimal size of bacterial aggregates. (**C**) Observation by confocal microscopy of *Lm* EGD ActA+ and ΔC+ mutants within stools of 96 h-infected mice. *Lm* was labeled with anti-*Lm* (red) and nuclei with Hoechst (blue). Scale: 2 µm. (**D**) Colonization assay performed on mice orally infected with EGD WT, Δ*actA*, ActA+ and ΔC+ strains. Each curve represented the mean total CFUs number obtained in stools, daily, from four different mice per bacterial strain. (**E**) Total number of bacteria shed from day 2 to the end of the previous colonization assay.

After four days of infection, ActA+ and ΔC+ *Lm* were eliminated from the small intestine lumen of infected mice (data not shown). In contrast, within the cecum lumen, we detected ActA+ bacteria forming aggregates, while ΔC+ bacteria remained essentially isolated in the lumen (Figure 7A). Indeed, the proportion of bacterial aggregates of more than three bacteria was four-fold higher in the cecum lumen of mice inoculated with ActA+ compared to ΔC+ bacteria (p<10^−6^) (Figure 7B). Bacterial aggregates were also detected within stools of mice inoculated with ActA+, whereas only rare and sparse bacteria were detected within stools of ΔC+-inoculated mice (Figure 7C). These results were confirmed using KI E16P mice inoculated with EGDe ActA+/ΔC+ (Figure S3A). Together, these results strongly suggest that the cecum is the site where *Lm* forms bacterial aggregates.

### ActA-dependent aggregation favors long-term persistence within gut lumen

Having shown that *Lm* aggregates within the cecum and colon lumens, we investigated whether *Lm* intraluminal aggregation might favor its persistence in the gut and fecal shedding. We inoculated KI E16P mice with EGD WT, Δ*actA*, ActA+ and ΔC+ bacteria and monitored *Lm* fecal carriage by enumerating daily bacterial CFUs in stools. Within the first two days, we observed the elimination of the bulk of the inoculum [44]. Fecal shedding of Δ*actA* and ΔC+ bacteria dropped steadily from day 1 and was no longer detectable after day 8 (Figure 7D). In sharp contrast, both WT and ActA+ bacteria showed increased fecal shedding between days 2 and 6, followed by a gradual and slow decline to finally reach total clearance by day 17 (Figure 7D). Indeed, total fecal shedding of *Lm* from day 2 to clearance was three orders of magnitude higher and persisted for twice as long in mice inoculated with WT or ActA+ *Lm* relative to mice inoculated with Δ*actA* or ΔC+ (Figure 7D–E). Similar results were observed when KI E16P mice were inoculated with EGDe ActA+/ΔC+ (Figures S3C–E), LO28 ActA+/ΔC+ (data not shown), and the PrfA+/PrfA− isolates (Figures S3B–D), which respectively express or not ActA (Figure S2A). These results show that even though ActA+ and ΔC+ bacteria invade mouse tissues at similar levels, their ability to colonize and persist the gut lumen strongly differs, illustrating that aggregating *Lm* display increased colonization and persistence in the gut than non-aggregating bacteria. This indicates that ActA, independent of its well-established role in bacterial dissemination within tissues in the systemic phase of the infection, also plays a critical role in intestinal colonization and long-term carriage of *Lm* within the gut.

## Discussion

*Lm* is adapted to survive in various conditions, colonize diverse environments, notably as a biofilm. It is also a facultative intracellular pathogen able to invade tissues and trigger a systemic infection in human and a wide range of animals. These two complementary aspects of *Lm* biology have so far been considered separately. We show here that, independently of its contribution to *Lm* actin-based motility that manifests intracellularly, ActA mediates *Lm* aggregation, colonization and persistence in the gut lumen, leading to its increased dissemination in the environment. To our knowledge, this is the first time that a virulence factor is involved in microbial persistence and transmission, independently of its known role in pathogenesis, This new property of ActA that occurs when *Lm* is located outside of the host cell, may apply a positive selective pressure for the maintenance of its gene, during the extracellular phase of its life cycle.

While we were studying this novel and unexpected function of ActA, two different investigators reported on the implication of PrfA in biofilm formation [27], [28], a process involving bacterial aggregation [30]. We show here that this process depends on ActA expression, which mediates inter-bacteria interactions and promotes biofilm formation. We also observed minor modulation of biofilm formation by two others PrfA-regulated factor, LLO and InlA, which slightly promotes and reduces *Lm* biofilm formation, respectively. Although the Δ*prfA* and Δ*actA* deletion mutants aggregation and biofilm phenotypes are indistinguishable, demonstrating that *actA* is the main PrfA-regulated gene accounting for the PrfA-dependence of *Lm* aggregation and biofilm formation, the contribution of InlA to *Lm* biofilm is in agreement with a previous study that showed that *inlA* mutations leading to InlA truncation slightly increase biofilm formation [47].

Studies in reference strains such as LO28 and EGDe have shown that ActA is up-regulated by PrfA when *Lm* is within the cytosol, in which ActA mediates actin-based motility [48]. ActA is also expressed in bacteria cultured in BHI liquid medium and within the gut lumen, although to a lower level than intracellularly (Figure S2C and [14]). Our initial observation of *Lm* aggregation was made in EGD, a reference strain that overexpresses ActA as a result of a gain-of-function mutation in *prfA* called prfA* (our unpublished data). We show here that in EGD, as well as in reference strains EGDe and LO28, ActA expression in BHI is sufficient to promote bacterial aggregation *in vitro*. This newly discovered property of ActA occurs at neutral pH and 37°C, the physiological environment of mammalian gut. In contrast, no aggregation is observed when bacteria are grown at 25°C, when PrfA-regulated genes are off, suggesting that ActA-dependent aggregation may contribute to *Lm* persistence in warm-blooded hosts (see below).

We demonstrate that *Lm* aggregation involves direct ActA-ActA interaction. We consistently observed that ActA-dependent aggregation occurs in PBS and H_2_O, suggesting that ActA-dependent aggregation might implicate direct ActA-ActA interaction. Consistent with this finding, SEM showed that *Lm* ActA-dependent aggregates do not contain detectable matrix or fiber-like material. Previous studies have shown that *Lm* ActA-dependent actin based motility relies on ActA polar distribution [49]. However, SEM on bacteria aggregates did not reveal any particular polar or lateral orientation in ActA-dependent bacterial interactions, which rather appeared to occur randomly. This suggests that in contrast to ActA-dependent actin-based motility, the polar distribution of ActA is not critical for *Lm* aggregation. We also show that the domain involved in ActA dimerization contributes to aggregation, indicating that ActA ability to dimerize might be implicated in the trans-dimerization of ActA molecules expressed by neighboring bacteria [50]. Yet, as for ActA dimerization, this domain is not sufficient to mediate bacterial aggregation. ActA is a particularly elongated molecule, largely made of random coils, which structure is responsible for many of its unique biochemical properties [51]. Although its three-dimensional structure is unknown, our results show that aggregation requires all ActA structural domains, suggesting that the native conformation of the protein is critical for aggregation. We have shown that ActA mediates *Lm* aggregation only above its pI, suggesting that ionic interactions between charged amino acids are essential in ActA-ActA interaction. ActA contains a particularly large amount of charged amino acids, especially within the 126–231 domain that is critical for aggregation. Because of its low pI (4.95), ActA is strongly charged at neutral pH, with a mix of positively and negatively charged regions likely involved in ActA-ActA mediated aggregation. However, ActA ortholog in *L. ivanovii*, IActA, which also mediates actin polymerization, does not mediate bacterial aggregation (our unpublished data), despite an identical pI, 34% of sequence identity and 52% of sequence similarity with ActA [52]. This suggests that ActA ability to mediate aggregation, although likely dependent on its charged residues, is a specific property of *Lm*. The sequence variability of *actA* has been used for typing purposes, and several studies have reported a high degree of polymorphism within *actA* [53]. Interestingly, a 105 bp deletion within *actA* region encoding the central proline rich repeat is frequently found in *Lm* [54]. As this deletion does not affect ActA ability to polymerize actin [55], we hypothesized that it may modify bacterial aggregation. However, we detected no significant association between aggregation ability of strains harboring or not this deletion (data not shown). We showed that the N-, P- and C-domains of ActA are critical for bacterial aggregation. Importantly, a mutant lacking C-region is still fully virulent. We took advantage of this property of the ActA C-domain to study specifically the role of ActA-dependent aggregation *in vivo*, independently of ActA contribution to actin polymerization. This led us to discover that the ability to form aggregate is associated to increased gut colonization and fecal shedding. To our knowledge, our study is the first demonstrating the involvement of a virulence factor in gut colonization and transmission that is independent of the mechanism mediating virulence. Indeed, although factors involved in gut colonization have been described for enteropathogenic bacteria such as *Salmonella* [56], enteropathogenic and enterohaemorrhagic *E. coli* [57], *Citrobacter rodentium* [58] and *Campylobacter jejuni* [59], in all cases, these effects were directly linked to their enteropathogenicity.

We have shown that *Lm*, when able to aggregate *in vitro*, also forms aggregates in the cecum and colon lumen, and colonizes the gut far more efficiently and durably than when it does not form aggregates. *Lm* is found in higher numbers in the cecum lumen than upstream in the small intestinal lumen [44], [60]. Furthermore, the gastric pH is highly acidic (1 to 2.5), whereas the pH varies between 6.4 and 7.5 from the small intestine to the cecum and colon. As ActA-mediated aggregation occurs between pH 6.5 to pH 9, *Lm* is subjected to a pH permissive to ActA-dependent aggregation in the distal small intestine, cecum and colon lumens but not within the stomach or the proximal duodenum lumens, which luminal content is far too acidic for ActA-mediated aggregation to occur. This hypothesis could not be verified as ingested bacteria were rapidly eliminated from small intestine lumen. The cecum lumen is likely the best site for aggregates formation: not only its greater diameter than the small intestine results in decreased shear stress, but also the increased number of intraluminal bacteria [4], [60] likely favors inter-bacterial contacts and hence aggregates formation. Aggregates observed within the cecum and colon lumens appeared to be mainly trapped within mucus whereas isolated bacteria were not, suggesting that mucus may favor *Lm* aggregate formation and/or expansion in the gut.

ActA has been shown to be expressed before intestinal tissue invasion, within the intestinal lumen [14] but the significance of this somewhat premature expression remained unexplained so far, as the role of ActA was thought to be exclusively intracellular. Here, we show that this extracellular expression of ActA allows intraluminal ActA-dependent aggregation, a property that correlates with increased gut colonization and fecal shedding. The release of *Lm* aggregates, as opposed to isolated bacteria, may favor *Lm* survival in environment and its transmission to new hosts, including animals and humans [61], [62]. It should be noted however that *Lm* virulence and particularly its ability to cross the intestinal barrier and survive in host tissues also affects its ability to colonize the gut: Δ*inlA* or Δ*hly* mutants for which virulence is attenuated *in vivo* also exhibit a reduced persistence in the intestine (data not shown). This suggests, as it has been recently proposed [44], that bacteria are shed back from infected intestinal villi into the intestinal lumen.

Among virulent *Listeria* species, *Lm* is the most prevalent species harboring *prfA* [63] and *Lm* is also the most prevalent species infecting mammalian hosts [64]. We demonstrate here that ActA favors long-term gut colonization and fecal shedding and that this advantage is *Lm*-specific. How and under which selective pressure has *Lm* acquired and evolved *prfA* and PrfA-regulated genes is not known. Virulence factors are thought to have been selected for as they allow pathogens to colonize otherwise sterile sites. Yet, the fact that ActA mediates *Lm* aggregation and intestinal colonization may have also participated the selective pressure on *Lm* to maintain ActA, as it favors *Lm* release in the environment and access to new hosts.

## Materials and Methods

### Bacterial strains and culture conditions

Bacterial strains used in this study are listed in Table S1. *Lm*, *Li* and *S. aureus* bacteria were cultured in Brain Heart Infusion medium (BHI, Difco) or in Dulbecco's Modified Eagle Medium (DMEM, Invitrogen), when specified. *E. coli* was cultivated in Luria Broth medium. Antibiotics were added when required at the following concentrations: erythromycin 5 µg/ml (*Li*) or 1 µg/ml (*S. aureus*) and chloramphenicol (Cm) 7 µg/ml (*Lm*) or 35 µg/ml (*E. coli*).

### Plasmids and strains construction

EGD Δ*prfA* and EGDe Δ*actA* mutants were constructed as previously described [65] using primers listed in Table S2. Stable insertion of Cm resistance gene in PrfA+ isolates, of GFP in EGD Δ*actA*, as well as chromosomal complementation of EGD Δ*actA*, EGDe Δ*actA* and LO28 Δ*actA* with full-length *actA* (ActA+) or *actA*ΔC (ΔC+) and EGD Δ*prfA* with *prfA* were realized as previously described [66] using plasmids pPL2, pAD cGFP, pPL2-*actA*, pPL2-*actA*ΔC and pPL2-*prfA*, respectively. The pPL2-*actA*, pPL2-*actA*ΔC and pPL2-*prfA* plasmids were constructed by PCR amplification from EGD chromosomal DNA of either full-length *actA*, *actA*ΔC and full-length *prfA* using primers listed in Table S2. These PCR fragments were cloned into pPL2 plasmid [67]. EGDe Δ*prfA*, LO28 Tn::*prfA*, *S. aureus* and *S. aureus* + *actA* were complemented after electroporation [68] of pMK4-*prfA* [12], pAT18 [2] or pAT18-*actA* [69] plasmids.

### Biofilm assays

#### Microtiter-plate biofilms

Exponential phase cultures were adjusted to OD_600_ of 0.05 in 96-well polyvinyl chloride microtiter plates (Falcon). Biofilms were let to grow 24 h at 37°C, fixed for 20 min in 50% Bouin's solution (Sigma Aldrich), washed in Phosphate Buffer Saline (PBS, Invitrogen) and stained with 10% crystal violet solution. Biomass stained was quantified by densitometry using Photoshop (Adobe) and ImageJ (National Institutes of Health) softwares.

#### Continuous-flow microfermentors biofilms

Microfermentors containing a removable glass spatula were used as described in (http://www.pasteur.fr/recherche/unites/Ggb/biofilmfermenter.html) to maximize biofilm development and minimize planktonic growth. Inoculation was performed by dipping the glass spatula for 2 min in culture adjusted to OD_600_ of 2. The spatula was then reintroduced into the microfermentor. After 42 h at 37°C, spatula was removed and biomass on spatula was resuspended in PBS. The final OD_600_ and the total number of *Lm* CFUs were measured.

#### Biofilms on static glass slide

Glass slides within Petri dishes were covered by exponential phase culture adjusted to OD_600_ of 0.05. Biofilms were let to grow in static condition 24 h at 37°C, and were carefully washed in 100 mM sodium cacodylate before fixation in 2.5% glutaraldehyde (Sigma Aldrich), 100 mM sodium cacodylate. Fixed biofilms were observed using LSM700 (Carl Zeiss) confocal microscope with a 10× water immersion objective. Three-dimensional reconstructions were performed using Imaris 5.5.3 software (Bitplane). Biofilm images were acquired with LSM 5 image browser (Carl Zeiss) and analyzed quantitatively using the Icy software (http://icy.bioimageanalysis.org). Each 3D stack was first filtered to increase the Signal-to-Noise Ratio. Then, an optimal intensity threshold between background and bacterium fluorescence levels was determined using a KMeans approach. Finally, the number and the volume of the connected fluorescent pixels defining the so-called clusters were calculated from the thresholded stack. The amount of bacteria per cluster was finally computed by dividing the volume of the each component by that of a single bacterium.

### Bacterial aggregation assay

Aggregation assay was performed in BHI, or in PBS after culture in BHI, for the strains in EGD genetic background, *Li* strain and for mutants in LO28 or EGDe background, when specified. Aggregation assay was realized in DMEM for the LO28 or EGDe background strains, for the strains from the NRC and for *S. aureus* strains, to induce higher expression of *actA* [31]. Stationary phase cultures were adjusted to the OD_600_ of 3 and let in static condition at 37°C up to 24 h. 75 µl samples were regularly taken from each sample, approximately 1 cm from the top to measure OD_600_ over time [29] and the so-called “aggregation in 24 h” was calculated by subtracting OD_600_ at 24 h to the initial OD_600_. After aggregation assay, bacteria that reached the bottom of the tubes were carefully collected and fixed on poly-L-lysine coated slides with 2.5% glutaraldehyde-100 mM sodium cacodylate. Fixed bacteria were observed by bright field microscopy or analyzed by SEM or immunofluorescence microscopy.

### Purification of ActA_HIS_, InlB_HIS_ and latex beads aggregation assay

Purification of ActA_HIS_ and InlB_HIS_ were performed as previously described [32], [34]. 50 µg of purified ActA_HIS_, InlB_HIS_ or BSA were coupled to 2 ml of 0.5% 1.1 µm polystyrene latex beads (Sigma Aldrich), in PBS and were let in static condition at 25°C up to 24 h. Samples of coupled latex beads were fixed on poly-L-lysine coated slides with 4% paraformaldehyde (Electron Microscopy Sciences) in PBS. Fixed beads were analyzed by immunofluorescence microscopy.

### Cellular invasion assay

T84 human intestinal cells (ATCC-CCL248) grown onto coverslips were washed in F12-DMEM (Invitrogen), kept at 4°C for 20 min, and incubated with bacteria (8.10^6^ bacteria/ml/well or multiplicity of infection of 100 per T84 cell). To synchronize *Lm* entry, bacteria were centrifugated at 200 g for 1 min at 4°C and incubated at 37°C with 5% CO_2_ for 40 min. T84 were washed to remove extracellular bacteria and were incubated 5 h at 37°C with 10 µg/ml gentamicin. After 5 h, cells and intracellular bacteria were washed, fixed with 4% paraformaldehyde and analyzed by immunofluorescence microscopy.

### Animals

Animal experiments with knock-in humanized E16P mEcad homozygous mice (KI E16P) permissive to InlA-Ecad interaction and orally-acquired listeriosis [4], were performed according to the Institut Pasteur guidelines for laboratory animals' husbandry. For oral infection, 8–12-week-old mice were fasted for 16 h before infection. After mild anesthesia of mice with 2.5% (vol/vol) vaporous isoflurane (Aerrane; Baxter), mice were orally infected with 5.10^9^ EGD bacteria or 10^9^ EGDe bacteria or 2.10^10^ LO28 bacteria or with 10^8^ bacteria of a mix of NRC isolates (CmR-PrfA+ and PrfA− isolates), as previously described [70]. At the planned endpoint (6 h or 96 h), the animal was euthanized, and spleen, liver, mesenteric lymph nodes, intestine and colon were collected. Before enumeration of CFUs, both intestines and colons were opened longitudinally, washed in DMEM and incubated under mild agitation with 100 µg/ml gentamicin to kill extracellular bacteria. CFUs within organs were enumerated as previously described [70]. To perform immunofluorescence microscopy on tissues, whole intestines, cecums and colons were collected and fixed with 4% paraformaldehyde, without any wash, and incubated in 30% sucrose. Organs were embedded in OCT and freeze before performing thin cryosections, as previously described [70]. For colonization assay, total mice stools were daily collected, weighed and resuspended in PBS before homogenization and enumeration of CFUs on *Listeria* selective Oxford medium (Oxoid). To discriminate CmR-PrfA+ bacteria from PrfA− ones within stools of mice inoculated with the mix of NRC isolates, *Lm* colonies grown on Oxford medium were then plated on BHI supplemented with Cm. Animals were euthanized when *Lm* was no more retrieved in the stools. Stools were fixed with 4% paraformaldehyde and analyzed by immunofluorescence microscopy.

To analyze ActA expression level within gut lumen, germ-free KI E16P mice [71] were inoculated with EGD, EGDe and LO28, as described above and euthanized 24 h or 96 h after inoculation. Feces were collected within cecums and colons, CFUs within feces were enumerated and ActA expression was analyzed by immunoblot.

All the procedures were in agreement with the guidelines of the European Commission for the handling of laboratory animals, directive 86/609/EEC (http://ec.europa.eu/environment/chemicals/lab_animals/home_en.htm) and were approved by the Animal Care and Use Committee of the Institut Pasteur.

### Scanning electron microscopy (SEM)

Bacteria from aggregation assay were washed in 0.2 M sodium cacodylate, fixed for 1 h in 1% osmium tetroxide in 0.2 M sodium cacodylate and then rinsed with distilled water. Samples were dehydrated through a graded series of 25, 50, 75 and 95% ethanol solution for 5 min. Samples were then dehydrated for 10 min in 100% ethanol followed by critical point drying with CO_2_. Dried specimens were sputtered with 10 nm gold palladium, with a GATAN Ion Beam Coater and were examined with a JEOL JSM 6700F field emission scanning electron microscope operating at 5Kv. Images were acquired with the lower secondary detector (LEI).

### Immunofluorescence microscopy

Fixed coupled latex beads were incubated without blocking step with monoclonal anti-HIS antibody (Sigma Aldrich) and Alexa-555 Fluor goat anti-mouse antibody (Invitrogen), in PBS. For all other immunolabeling assays, samples were blocked in blocking buffer (PBS, 4% BSA) for 20 min, and then maintained in blocking conditions during all the staining steps. Cells permeabilization was performed in 0.3% Triton X-100. Bacteria were labeled with rabbit polyclonal antibodies anti-*Lm* R11 [72] and the Alexa-555 Fluor goat anti-rabbit antibody (Invitrogen). Nuclei were detected using the DNA marker Hoechst 33342 (Invitrogen). T84 cells containing bacteria were labeled with monoclonal mouse anti-human E-cadherin HECD-1 (Invitrogen) and Alexa Fluor 647 goat anti-mouse (Invitrogen). Cells, as well as actin tails, were highlighted with Alexa Fluor 488 phalloidin (Invitrogen). E-cadherin on mice tissues was detected with monoclonal rat anti-ECCD-2 (Invitrogen) and Alexa Fluor 488 goat anti-rat (Invitrogen). Wheat germ agglutinin (WGA), Alexa Fluor 647 conjugate (Invitrogen) was used to label goblet cells, basal membranes and mucus [6]. Samples were observed with an AxioObserver microscope (Carl Zeiss) or with a LSM700 confocal microscope. Pictures and Z-stacks were acquired using AxioVision 4.5 or LSM 5 image browser softwares. From T84 acquired images, percentage of intracellular bacteria harboring an actin tail was quantified among 1500 to 2000 intracellular bacteria using ImageJ software, and length of the comet tails was measured using AxioVision 4.5 software. From acquired images of mice cecum lumen, aggregating bacteria (at least three interacting bacteria) were counted among 1000 to 2000 bacteria using ImageJ software.

### Protein analysis

To analyze ActA expression in EGD, EGDe, LO28, *Li* + *actA*, in NRC isolates and in ActA-truncated mutants, stationary phase cultures were pelleted and resuspended in order to load equivalent of 0.2 OD_600_ bacteria per well. ActA expression in gut lumen was analyzed by loading the equivalent of 10^7^ bacteria within gut feces. Denaturated samples were separated on SDS-PAGE gels (Biorad) and transferred on PVDF membrane (GE Healthcare) to perform immunoblot. ActA was detected using affinity-purified polyclonal ActA-specific antibodies P473 (P102–123) [73], ActA-truncated regions were revealed using affinity-purified polyclonal ActA-specific antibodies A18K [73] and amount of loaded bacteria was checked using affinity-purified polyclonal EF-Tu-specific antibodies R-114 [74], all revealed using peroxidase-coupled anti-rabbit antibody (GE Healthcare). The PVDF membranes were developed by enhanced chemiluminescence using ECL (Amersham). Protein levels were quantified by measuring the intensity of the bands by densitometry using Photoshop and ActA protein level was normalized with EF-Tu level.

### Statistical analysis

Each experiment was realized at least three times. Within each experiment, means were calculated from at least three samples. Student's t tests were performed for all experiments, except for comparison of *in vivo* virulence and colonization for which Mann-Whitney tests were performed. The level of significance is shown in each figure (NS p>0.05, * p≤0.05, ** p≤0.01 and *** p≤0.005).

## Supporting Information

Figure S1 ***actA*** **, a PrfA-regulated gene, mediates LO28 and EGDe aggregation.** (**A**) Results of aggregation assays performed on LO28 WT strain and LO28 Tn::*prfA* in BHI. (**B**) Results of aggregation assays performed on EGDe WT strain and EGDe Δ*prfA* in BHI. (**C**) Results of aggregation assays performed on LO28 WT strain and LO28 Tn::*prfA* in DMEM to increase PrfA-regulated genes expression and LO28 aggregation. (**D**) Results of aggregation assays performed on EGDe WT strain and EGDe Δ*prfA* in DMEM. (**E**) Results of aggregation assays in DMEM of LO28 WT, LO28 Tn::*prfA*, the complemented mutant LO28 Tn::*prfA* + *actA*, LO28 Δ*actA* and the complemented strain LO28 Δ*actA + actA*. (**F**) Results of aggregation assays in DMEM of EGDe WT, EGDe Δ*prfA*, the complemented mutants EGDe Δ*prfA* + *prfA* and EGDe Δ*prfA + actA*, LO28 Δ*actA* and the complemented strain LO28 Δ*actA + actA*.
(TIF)

Figure S2 **ActA expression analysis.** (**A**) Immunoblot performed on NRC strains including the PrfA+ isolates and the PrfA− isolates. ActA was revealed using the anti-ActA P473 antibody and the amount of loaded bacteria was controlled using the anti-EF-Tu R-114. (**B**) Immunoblot performed on LO28 truncated mutants of ActA. ActA was revealed using the anti-ActA A18K antibody. (**C**) Comparison of ActA expression levels in the *Lm* strains EGD, EGD Δ*actA*, LO28 and EGDe in stationary phase BHI culture and within the mice cecum-colon lumen. ActA intensity signal revealed by immunoblot was quantified by densitometry and normalized with EF-Tu intensity signal.
(TIF)

Figure S3 **ActA promotes clinical isolates and EGDe aggregation within gut lumen and favors intestinal colonization.** (**A**) Imaging of EGDe Δ*actA* + *actA* (EGDe ActA+) and EGDe Δ*actA* + *actA*ΔC (EGDe ΔC+) mutants within stools of 96 h-infected mice. *Lm* was labeled with anti-*Lm* (red) and nuclei with Hoechst (blue). Scale: 2 µm. (**B**) Colonization assay performed on eight mice orally infected with the same mix of PrfA+/PrfA− NRC isolates. For each mouse, CFUs were daily enumerated within collected stools and ratio of chloramphenicol (Cm)-resistant PrfA+ bacteria was calculated by duplicating CFUs on Cm plates. Each curve represented the mean total CFUs number of PrfA+ *versus* PrfA− obtained from the eight mice. (**C**) Colonization assay performed on mice orally infected with EGDe Δ*actA* + *actA* (EGDe ActA+) and EGDe Δ*actA* + *actA*ΔC (EGDe ΔC+) strains. Each curve represented the mean total CFUs number obtained in stools, daily, from six different mice per bacterial strain. (**D**) Total number of bacteria shed from day 2 to the end of the colonization assay in (B). (**E**) Total number of bacteria shed from day 2 to the end of the colonization assay in (C).
(TIF)

Table S1 **Strains used in this study.** All the strains used in the study are listed and referenced. Plasmids used for mutant complementation are cited and also references. Origin of the complementation genes, as well as the promoter and the ribosome-binding site allowing their expression, are noted. CmR: chloramphenicol-resistant.
(DOC)

Table S2 **Primers used in this study.** Primers used for construction of EGD Δ*prfA* (*prfA*-1-F, *prfA*-1-R, *prfA*-2-F and *prfA*-2-R), EGD Δ*prfA* + *prfA* (*prfA*-5 and *prfA*-3), EGDe Δ*actA* (*actA*-L1, *actA*-R1, *actA*-L2 and *actA*-R2), EGD Δ*actA* + *actA* (*actA*-5-F and *actA*-200-3-R) and EGD Δ*actA* + *actA*ΔC (*actA*-5-F, *actA*-AA441-3, *actA*-AA608-5 and *actA*-200-3-R) are listed.
(DOC)
