# Supplementary figures and images for: ActA Promotes Listeria monocytogenes Aggregation, Intestinal Colonization and Carriage
Source: PLoS Pathog. 2013 Jan 31;9(1):e1003131. doi: 10.1371/journal.ppat.1003131 (PMC3561219; doi:10.1371/journal.ppat.1003131)

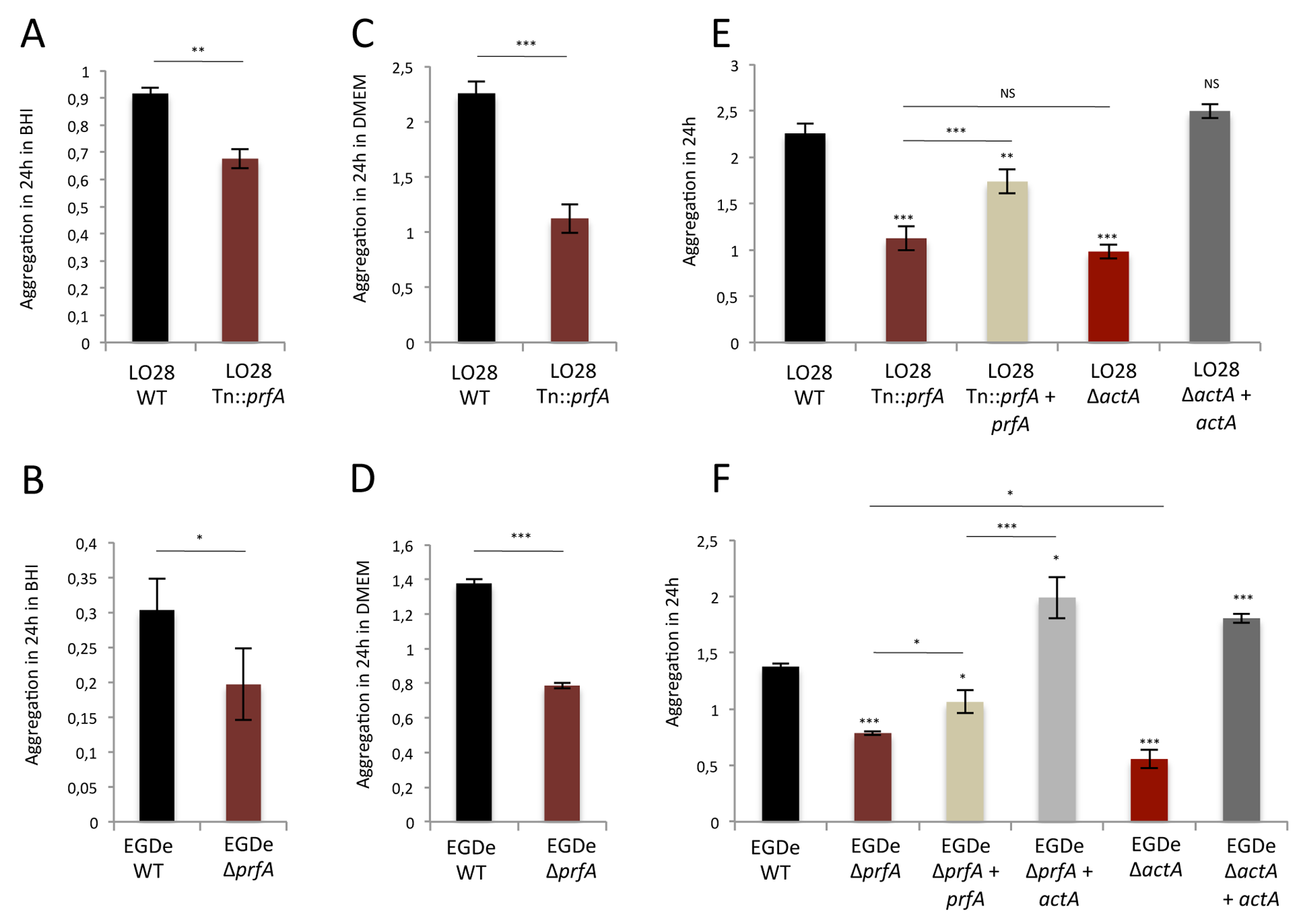

Supplement: Figure S1 — actA , a PrfA-regulated gene, mediates LO28 and EGDe aggregation. (A) Results of aggregation assays performed on LO28 WT strain and LO28 Tn::prfA in BHI. (B) Results of aggregation assays performed on EGDe WT strain and EGDe ΔprfA in BHI. (C) Results of aggregation assays performed on LO28 WT strain and LO28 Tn::prfA in DMEM to increase PrfA-regulated genes expression and LO28 aggregation. (D) Results of aggregation assays performed on EGDe WT strain and EGDe ΔprfA in DMEM. (E) Results of aggregation assays in DMEM of LO28 WT, LO28 Tn::prfA, the complemented mutant LO28 Tn::prfA + actA, LO28 ΔactA and the complemented strain LO28 ΔactA + actA. (F) Results of aggregation assays in DMEM of EGDe WT, EGDe ΔprfA, the complemented mutants EGDe ΔprfA + prfA and EGDe ΔprfA + actA, LO28 ΔactA and the complemented strain LO28 ΔactA + actA. (TIF) [file ppat.1003131.s001.tif]

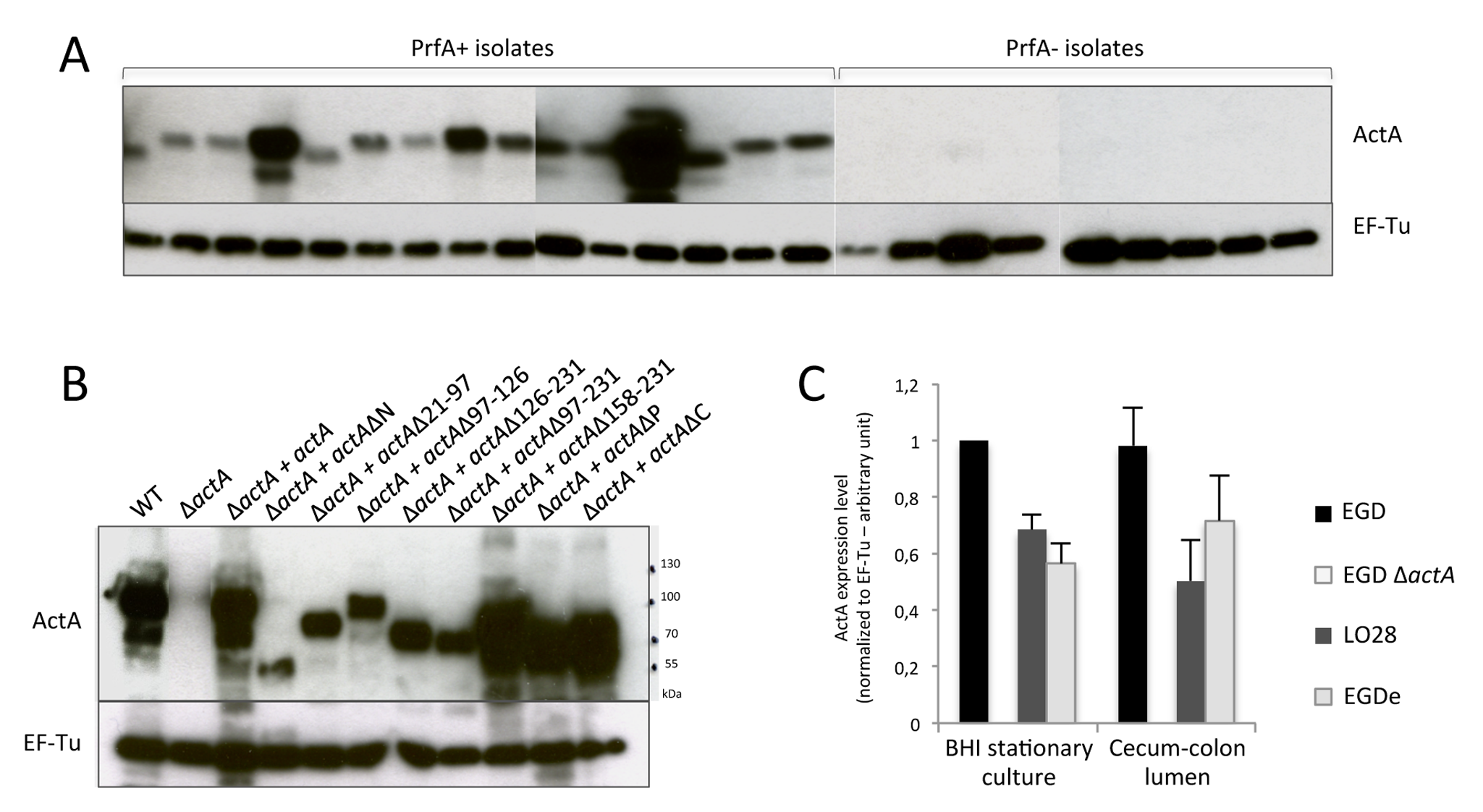

Supplement: Figure S2 — ActA expression analysis. (A) Immunoblot performed on NRC strains including the PrfA+ isolates and the PrfA− isolates. ActA was revealed using the anti-ActA P473 antibody and the amount of loaded bacteria was controlled using the anti-EF-Tu R-114. (B) Immunoblot performed on LO28 truncated mutants of ActA. ActA was revealed using the anti-ActA A18K antibody. (C) Comparison of ActA expression levels in the Lm strains EGD, EGD ΔactA, LO28 and EGDe in stationary phase BHI culture and within the mice cecum-colon lumen. ActA intensity signal revealed by immunoblot was quantified by densitometry and normalized with EF-Tu intensity signal. (TIF) [file ppat.1003131.s002.tif]

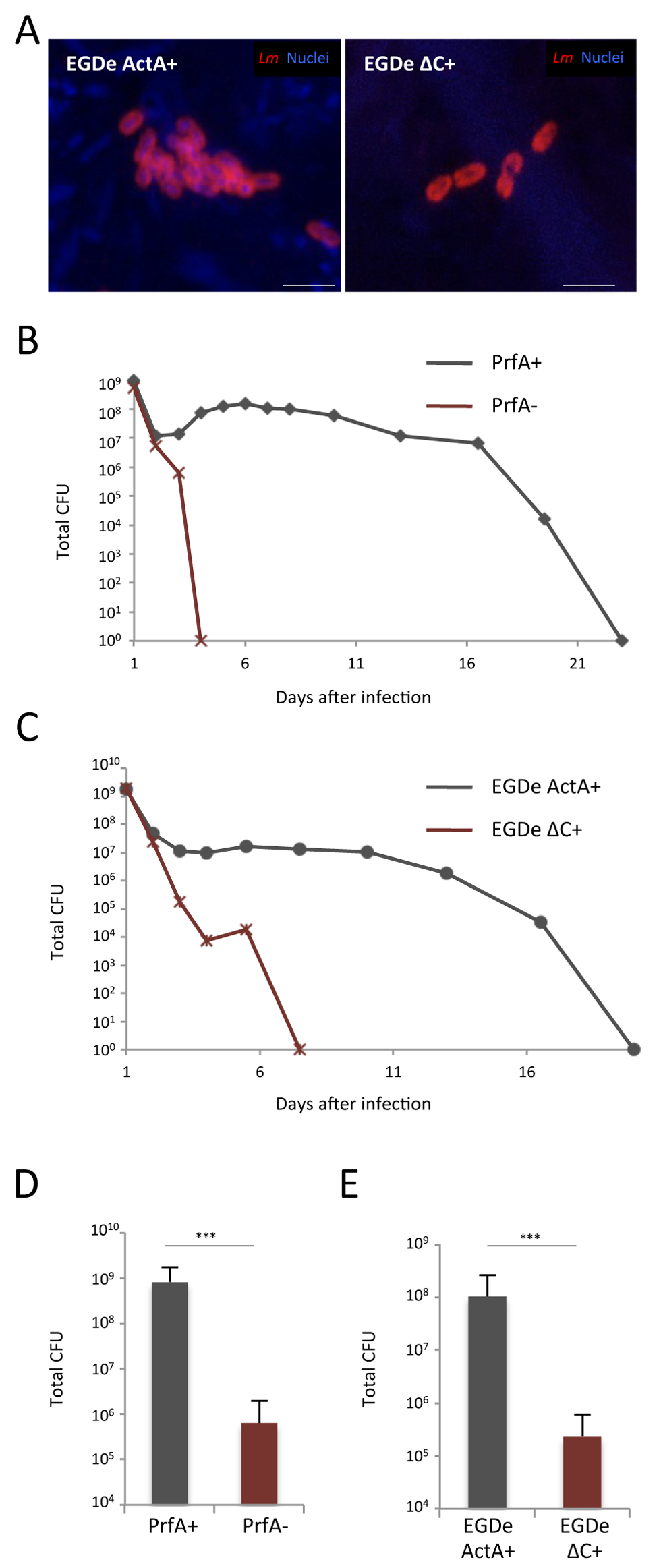

Supplement: Figure S3 — ActA promotes clinical isolates and EGDe aggregation within gut lumen and favors intestinal colonization. (A) Imaging of EGDe ΔactA + actA (EGDe ActA+) and EGDe ΔactA + actAΔC (EGDe ΔC+) mutants within stools of 96 h-infected mice. Lm was labeled with anti-Lm (red) and nuclei with Hoechst (blue). Scale: 2 µm. (B) Colonization assay performed on eight mice orally infected with the same mix of PrfA+/PrfA− NRC isolates. For each mouse, CFUs were daily enumerated within collected stools and ratio of chloramphenicol (Cm)-resistant PrfA+ bacteria was calculated by duplicating CFUs on Cm plates. Each curve represented the mean total CFUs number of PrfA+ versus PrfA− obtained from the eight mice. (C) Colonization assay performed on mice orally infected with EGDe ΔactA + actA (EGDe ActA+) and EGDe ΔactA + actAΔC (EGDe ΔC+) strains. Each curve represented the mean total CFUs number obtained in stools, daily, from six different mice per bacterial strain. (D) Total number of bacteria shed from day 2 to the end of the colonization assay in (B). (E) Total number of bacteria shed from day 2 to the end of the colonization assay in (C). (TIF) [file ppat.1003131.s003.tif]
