# Supplementary material for: ActA Promotes Listeria monocytogenes Aggregation, Intestinal Colonization and Carriage
Source: PLoS Pathog. 2013 Jan 31;9(1):e1003131. doi: 10.1371/journal.ppat.1003131 (PMC3561219; doi:10.1371/journal.ppat.1003131)
Supplement: Table S1 — Strains used in this study. All the strains used in the study are listed and referenced. Plasmids used for mutant complementation are cited and also references. Origin of the complementation genes, as well as the promoter and the ribosome-binding site allowing their expression, are noted. CmR: chloramphenicol-resistant. (DOC) [file ppat.1003131.s004.doc]

**Table S1: Strains**

| **Strains** | **Construction - Characteristics** | **Origin - Source** |
| --- | --- | --- |
| EGD – BUG600 |  | [1] |
| EGD GFP – BUG2539 |  | [2] |
| EGD ∆*prfA* – BUG2141 |  | This study |
| EGD ∆*prfA* + *prfA* | EGD ∆*prfA* + pPL2-*prfA* (EGD promoter and rbs – this study) | This study |
| EGD ∆*prfA* + *actA* | EGD ∆*prfA +* pAT18-*actA* (constitutive expression, pPROT promoter and rbs [3]) [4] | This study |
| EGD ∆*inlA* – BUG947 |  | [5] |
| EGD ∆*inlB* – BUG1047 |  | [5] |
| EGD ∆*hly* – BUG2132 |  | [6] |
| EGD ∆*actA* – BUG2140 |  | [7] |
| EGD ∆*actA* GFP | EGD ∆*actA* + pAD cGFP plasmid [2] | This study |
| EGD ∆*actA* + *actA* | EGD ∆*actA* + pPL2-*actA* (EGD promoter and rbs – this study) | This study |
| EGD ∆*actA* + *actA*∆C | EGD ∆*actA* + pPL2-*actA*∆C (EGD promoter and rbs – this study) | This study |
| *L. innocua* – BUG994 | With empty pAT18 plasmid | [8] |
| *L. innocua* + *actA* – BUG852 | With pAT18-*actA* (constitutive expression, pPROT promoter and rbs [3]) [4] | [4] |
| *S. aureus* | *S. aureus* [ATCC 25923] with empty pAT18 plasmid [8] | This study |
| *S. aureus* + *actA* | *S. aureus* [ATCC 25923] with pAT18-*actA* (constitutive expression, pPROT promoter and rbs [3]) [4] | This study |
| *Lm* DPL1545 | With pDP2717 plasmid (secreted ActA_HIS_ AA1-613) | [9] |
| *E. coli* BL21-InlB_HIS_ | With pET28b1-InlB_HIS_ plasmid | [10] |
| EGDe – BUG1600 |  | [11] |
| EGDe ∆*prfA -* BUG2214 |  | [12] |
| EGDe ∆*prfA* + *prfA* | EGDe ∆*prfA* + pMK4-*prfA* [13] (LO28 promoter and rbs) | This study |
| EGDe ∆*prfA* + *actA* | EGDe ∆*prfA +* pAT18-*actA* (constitutive expression, pPROT promoter and rbs [3]) [4] | This study |
| EGDe ∆*actA* – BUG2167 |  | This study |
| EGDe ∆*actA* + *actA* | EGDe ∆*actA* + pPL2-*actA* (EGD promoter and rbs - this study) | This study |
| EGDe ∆*actA* + *actA*∆C | EGDe ∆*actA* + pPL2-*actA*∆C (EGD promoter and rbs - this study) | This study |
| LO28 – Bof343 |  | [14] |
| LO28 Tn::*prfA* – BUG597 |  | [13] |
| LO28 Tn::*prfA* + *prfA* | LO28 Tn::*prfA* + pMK4-*prfA* [13] (LO28 promoter and rbs) | [13] |
| LO28 ∆*actA* – BUG875 |  | [15] |
| LO28 ∆*actA* + *actA* – BUG666 | LO28 ∆*actA* + p*actA*-*actA* (LO28 promoter and rbs) | [16] |
| LO28 ∆*actA* + *actA*∆N – BUG1274 | LO28 ∆*actA* + p*actA*-*actA*∆N (LO28 promoter and rbs) | [17] |
| LO28 ∆*actA* + *actA*∆21-97 – BUG1376 | LO28 ∆*actA* + p*actA*-*actA*∆21-97 (LO28 promoter and rbs) | [18] |
| LO28 ∆*actA* + *actA*∆97-126 – BUG1539 | LO28 ∆*actA* + p*actA*-*actA*∆97-126 (LO28 promoter and rbs) | [16] |
| LO28 ∆*actA* + *actA*∆126-231 – BUG1370 | LO28 ∆*actA* + p*actA*-*actA*∆126-231 (LO28 promoter and rbs) | [18] |
| LO28 ∆*actA* + *actA*∆97-231 – BUG1374 | LO28 ∆*actA* + p*actA*-*actA*∆97-231 (LO28 promoter and rbs) | [18] |
| LO28 ∆*actA* + *actA*∆158-231 – BUG1368 | LO28 ∆*actA* + p*actA*-*actA*∆158-231 (LO28 promoter and rbs) | [18] |
| LO28 ∆*actA* + *actA*∆P – BUG1277 | LO28 ∆*actA* + p*actA*-*actA*∆P (LO28 promoter and rbs) | [17] |
| LO28 ∆*actA* + *actA*∆C – BUG1275 | LO28 ∆*actA* + p*actA*-*actA*∆C (LO28 promoter and rbs) | [17] |
| LO28 ∆*actA* + *actA* | LO28 ∆*actA* + pPL2-*actA* (EGD promoter and rbs – this study) | This study |
| LO28 ∆*actA* + *actA*∆C | LO28 ∆*actA* + pPL2-*actA*∆C (EGD promoter and rbs – this study) | This study |
| CLIP 2009/01337 | Clinical isolate | NRC and WHO-CC *Listeria* |
| CLIP 2009/01337 CmR | CLIP 2009/01337 + pPL2 [19] | This study |
| CLIP 2009/01341 | Clinical isolate | NRC and WHO-CC *Listeria* |
| CLIP 2009/01341 CmR | CLIP 2009/01341 + pPL2 [19] | This study |
| CLIP 2009/01351 | Clinical isolate | NRC and WHO-CC *Listeria* |
| CLIP 2009/01351 CmR | CLIP 2009/01351 + pPL2 [19] | This study |
| CLIP 2009/01377 | Clinical isolate | NRC and WHO-CC *Listeria* |
| CLIP 2009/01377 CmR | CLIP 2009/01377 + pPL2 [19] | This study |
| CLIP 2010/00009 | Clinical isolate | NRC and WHO-CC *Listeria* |
| CLIP 2010/00009 CmR | CLIP 2010/00009 + pPL2 [19] | This study |
| CLIP 2010/00095 | Clinical isolate | NRC and WHO-CC *Listeria* |
| CLIP 2010/00095 CmR | CLIP 2010/00095 + pPL2 [19] | This study |
| CLIP 2010/00112 | Clinical isolate | NRC and WHO-CC *Listeria* |
| CLIP 2010/00112 CmR | CLIP 2010/00112 + pPL2 [19] | This study |
| CLIP 2010/00133 | Clinical isolate | NRC and WHO-CC *Listeria* |
| CLIP 2010/00133 CmR | CLIP 2010/00133 + pPL2 [19] | This study |
| CLIP 2010/00184 | Clinical isolate | NRC and WHO-CC *Listeria* |
| CLIP 2010/00184 CmR | CLIP 2010/00184 + pPL2 [19] | This study |
| CLIP 2010/00241 | Clinical isolate | NRC and WHO-CC *Listeria* |
| CLIP 2010/00241 CmR | CLIP 2010/00241 + pPL2 [19] | This study |
| CLIP 2010/00403 | Clinical isolate | NRC and WHO-CC *Listeria* |
| CLIP 2010/00403 CmR | CLIP 2010/00403 + pPL2 [19] | This study |
| CLIP 2010/00435 | Clinical isolate | NRC and WHO-CC *Listeria* |
| CLIP 2010/00435 CmR | CLIP 2010/00435 + pPL2 [19] | This study |
| CLIP 2010/00518 | Clinical isolate | NRC and WHO-CC *Listeria* |
| CLIP 2010/00518 CmR | CLIP 2010/00518 + pPL2 [19] | This study |
| CLIP 2010/00562 | Clinical isolate | NRC and WHO-CC *Listeria* |
| CLIP 2010/00562 CmR | CLIP 2010/00562 + pPL2 [19] | This study |
| CLIP 2010/00605 | Clinical isolate | NRC and WHO-CC *Listeria* |
| CLIP 2010/00605 CmR | CLIP 2010/00605 + pPL2 [19] | This study |
| PrfA- isolate 1 | Non clinical isolate | NRC and WHO-CC *Listeria* |
| PrfA- isolate 2 | Non clinical isolate | NRC and WHO-CC *Listeria* |
| PrfA- isolate 3 | Non clinical isolate | NRC and WHO-CC *Listeria* |
| PrfA- isolate 4 | Non clinical isolate | NRC and WHO-CC *Listeria* |
| PrfA- isolate 5 | Non clinical isolate | NRC and WHO-CC *Listeria* |
| PrfA- isolate 6 | Non clinical isolate | NRC and WHO-CC *Listeria* |
| PrfA- isolate 7 | Non clinical isolate | NRC and WHO-CC *Listeria* |
| PrfA- isolate 8 | Non clinical isolate | NRC and WHO-CC *Listeria* |
| PrfA- isolate 9 | Non clinical isolate | NRC and WHO-CC *Listeria* |

1. Mengaud J, Geoffroy C, Cossart P (1991) Identification of a new operon involved in Listeria monocytogenes virulence: its first gene encodes a protein homologous to bacterial metalloproteases. Infect Immun 59: 1043-1049.

2. Balestrino D, Hamon MA, Dortet L, Nahori M-A, Pizarro-Cerda J, et al. (2010) Single-cell techniques using chromosomally tagged fluorescent bacteria to study Listeria monocytogenes infection processes. Applied and Environmental Microbiology 76: 3625-3636.

3. Kok J, Leenhouts KJ, Haandrikman AJ, Ledeboer AM, Venema G (1988) Nucleotide sequence of the cell wall proteinase gene of Streptococcus cremoris Wg2. Appl Environ Microbiol 54: 231-238.

4. Kocks C, Marchand JB, Gouin E, d'Hauteville H, Sansonetti PJ, et al. (1995) The unrelated surface proteins ActA of Listeria monocytogenes and IcsA of Shigella flexneri are sufficient to confer actin-based motility on Listeria innocua and Escherichia coli respectively. Molecular Microbiology 18: 413-423.

5. Dramsi S, Biswas I, Maguin E, Braun L, Mastroeni P, et al. (1995) Entry of Listeria monocytogenes into hepatocytes requires expression of inIB, a surface protein of the internalin multigene family. Molecular Microbiology 16: 251-261.

6. Hamon MA, Cossart P (2011) K+ efflux is required for histone H3 dephosphorylation by Listeria monocytogenes listeriolysin O and other pore-forming toxins. Infection and Immunity 79: 2839-2846.

7. Levraud J-P, Disson O, Kissa K, Bonne I, Cossart P, et al. (2009) Real-time observation of listeria monocytogenes-phagocyte interactions in living zebrafish larvae. Infection and Immunity 77: 3651-3660.

8. Gormley E, Mengaud J, Cossart P (1989) Sequences homologous to the listeriolysin O gene region of Listeria monocytogenes are present in virulent and avirulent haemolytic species of the genus Listeria. Res Microbiol 140: 631-643.

9. Welch MD, Rosenblatt J, Skoble J, Portnoy DA, Mitchison TJ (1998) Interaction of human Arp2/3 complex and the Listeria monocytogenes ActA protein in actin filament nucleation. Science 281: 105-108.

10. Braun L, Dramsi S, Dehoux P, Bierne H, Lindahl G, et al. (1997) InlB: an invasion protein of Listeria monocytogenes with a novel type of surface association. Molecular Microbiology 25: 285-294.

11. Glaser P, Frangeul L, Buchrieser C, Rusniok C, Amend A, et al. (2001) Comparative genomics of Listeria species. Science 294: 849-852.

12. Böckmann R, Dickneite C, Middendorf B, Goebel W, Sokolovic Z (1996) Specific binding of the Listeria monocytogenes transcriptional regulator PrfA to target sequences requires additional factor(s) and is influenced by iron. Mol Microbiol 22: 643-653.

13. Mengaud J, Dramsi S, Gouin E, Vazquez-Boland JA, Milon G, et al. (1991) Pleiotropic control of Listeria monocytogenes virulence factors by a gene that is autoregulated. Molecular Microbiology 5: 2273-2283.

14. Vicente MF, Baquero F, Cossart P, Pérez-Díaz JC (1987) Cloning of two possible haemolysin determinants from Listeria monocytogenes. Ann Inst Pasteur Microbiol 138: 385-387.

15. Gouin E, Dehoux P, Mengaud J, Kocks C, Cossart P (1995) iactA of Listeria ivanovii, although distantly related to Listeria monocytogenes actA, restores actin tail formation in an L. monocytogenes actA mutant. Infection and Immunity 63: 2729-2737.

16. Mourrain P, Lasa I, Gautreau A, Gouin E, Pugsley A, et al. (1997) ActA is a dimer. Proc Natl Acad Sci USA 94: 10034-10039.

17. Lasa I, David V, Gouin E, Marchand JB, Cossart P (1995) The amino-terminal part of ActA is critical for the actin-based motility of Listeria monocytogenes; the central proline-rich region acts as a stimulator. Molecular Microbiology 18: 425-436.

18. Lasa I, Gouin E, Goethals M, Vancompernolle K, David V, et al. (1997) Identification of two regions in the N-terminal domain of ActA involved in the actin comet tail formation by Listeria monocytogenes. EMBO J 16: 1531-1540.

19. Lauer P, Chow MYN, Loessner MJ, Portnoy DA, Calendar R (2002) Construction, characterization, and use of two Listeria monocytogenes site-specific phage integration vectors. Journal of Bacteriology 184: 4177-4186.
