# Supplementary material for: ActA Promotes Listeria monocytogenes Aggregation, Intestinal Colonization and Carriage
Source: PLoS Pathog. 2013 Jan 31;9(1):e1003131. doi: 10.1371/journal.ppat.1003131 (PMC3561219; doi:10.1371/journal.ppat.1003131)
Supplement: Table S2 — Primers used in this study. Primers used for construction of EGD ΔprfA (prfA-1-F, prfA-1-R, prfA-2-F and prfA-2-R), EGD ΔprfA + prfA (prfA-5 and prfA-3), EGDe ΔactA (actA-L1, actA-R1, actA-L2 and actA-R2), EGD ΔactA + actA (actA-5-F and actA-200-3-R) and EGD ΔactA + actAΔC (actA-5-F, actA-AA441-3, actA-AA608-5 and actA-200-3-R) are listed. (DOC) [file ppat.1003131.s005.doc]

Table S2: Primers

| **Primer**  **F: forward**  **R: reverse** | **Sequence** | **Amplification** |
| --- | --- | --- |
| *prfA*-1-F | 5'-gaagatctcctgacacaacaaacctagcagcgc-3' | Region upstream *prfA* gene for EGD ∆*prfA* construction |
| *prfA*-1-R | 5'-cggaattcgtctcatcatcccccaatcgttttttatcgc-3' | Region upstream *prfA* gene for EGD ∆*prfA* construction |
| *prfA*-2-F | 5'-cggaattcatcaaaaacagtattcctcaatgagg-3' | Region downstream *prfA* gene for EGD ∆*prfA* construction |
| *prfA*-2-R | 5'-cgggatcctcgtgaactagctgaagagattgcg-3' | Region downstream *prfA* gene for EGD ∆*prfA* construction |
| *prfA*-5 | 5'-caagcttccaaagcggacaataaac-3' | Region upstream *prfA* gene for EGD ∆*prfA* complementation |
| *prfA*-3 | 5'-acatcgttggtaatgtagaaggaa-3' | Region downstream *prfA* gene for EGD ∆*prfA* complementation |
| *actA*-L1 | 5’-gaagatctagtggatagaactcataaaggactc-3’ | Region upstream *actA* gene for EGDe ∆*actA* construction |
| *actA*-R1 | 5’-cggaattcttatactccctcctcgtgatacgc-3’ | Region upstream *actA* gene for EGDe ∆*actA* construction |
| *actA*-L2 | 5’-cggaattcaaacacagaacgaaagaaaaagtgaggtg-3’ | Region downstream *actA* gene for EGDe ∆*actA* construction |
| *actA*-R2 | 5’-cgggatccaacctcccttttatatacatttggc-3’ | Region downstream *actA* gene for EGDe ∆*actA* construction |
| *actA*-5-F | 5’-gattaacaaatgttagagaaaaattaattctcc-3’ | Full-length *actA* fragment for pPL2-*actA* construction  Region upstream ∆C-deletion and *actA*∆C fragment for pPL2-*actA*∆C construction |
| *actA*-200-3-R | 5’-atccgcggaccaactaagtttatg-3’ | Full-length *actA* fragment for pPL2-*actA* construction  Region downstream ∆C-deletion and *actA*∆C fragment for pPL2-*actA*∆C construction |
| *actA*-AA608-5 | 5’-gatttaagagatagaggaacaggaaaacactcaagaaatgcg  gggaaccatacgacgttaat-3’ | Region downstream ∆C-deletion for pPL2-*actA*∆C construction |
| *actA*-AA441-3 | 5’-ccaatagctaacattgcaagaattaacgtcgtatggttcccc  gcatttcttgagtgttttc-3’ | Region upstream ∆C-deletion for pPL2-*actA*∆C construction |
